# Autologous non-invasively derived stem cells mitochondria transfer shows therapeutic advantages in human embryo quality rescue

**DOI:** 10.1186/s40659-023-00470-1

**Published:** 2023-11-17

**Authors:** Zhixin Jiang, Cheng Shi, Hongjing Han, Min Fu, Honglan Zhu, Tingting Han, Jia Fei, Yining Huang, Zhiping Jin, Jianan He, Yanbin Wang, Xi Chen, Huan Shen

**Affiliations:** 1 Reproductive Medical Center, Peking University People’s Hospital, Peking University, Beijing, 100044 China; 2 Center for Reproductive Medicine, Ren Ji Hospital, School of Medicine, Shanghai Jiao Tong University, Shanghai, 200135 China; 3 Shanghai Key Laboratory for Assisted Reproduction and Reproductive Genetics, Shanghai, 200135 China; 4 Department of Obstetrics and Gynecology, Peking University People’s Hospital, Peking University, Beijing, 100044 China; 5 Peking University Institute of Hematology, National Clinical Research Center for Hematologic Disease, Beijing Key Laboratory of Hematopoietic Stem Cell Transplantation, Collaborative Innovation Center of Hematology, Peking University People’s Hospital, Peking University, Beijing, 100044 China; 6 Peking Jabrehoo Med Tech Co., Ltd, Beijing, 102629 China

**Keywords:** Infertility, Oocyte aging, Autologous mitochondria transfer, Embryo development, Mitochondrial metabolism, Urine-derived mesenchymal stromal cells

## Abstract

**Background:**

The decline in the quantity and quality of mitochondria are closely associated with infertility, particularly in advanced maternal age. Transferring autologous mitochondria into the oocytes of infertile females represents an innovative and viable strategy for treating infertility, with no concerns regarding ethical considerations. As the donor cells of mitochondria, stem cells have biological advantages but research and evidence in this area are quite scarce.

**Methods:**

To screen out suitable human autologous ooplasmic mitochondrial donor cells, we performed comprehensive assessment of mitochondrial physiology, function and metabolic capacity on a varity of autologous adipose, marrow, and urine-derived mesenchymal stromal cells (ADSC, BMSC and USC) and ovarian germline granulosa cells (GC). Further, to explore the biosafety, effect and mechanism of stem cell-derived mitochondria transfer on human early embryo development, randomized in-vitro basic studies were performed in both of the young and aged oocytes from infertile females.

**Results:**

Compared with other types of mesenchymal stromal cells, USC demonstrated a non-fused spherical mitochondrial morphology and low oxidative stress status which resembled the oocyte stage. Moreover, USC mitochondrial content, activity and function were all higher than other cell types and less affected by age, and it also exhibited a biphasic metabolic pattern similar to the pre-implantation stage of embryonic development. After the biosafety identification of the USC mitochondrial genome, early embryos after USC mitochondrial transfer showed improvements in mitochondrial content, activity, and cytoplasmic Ca^2+^ levels. Further, aging embryos also showed improvements in embryonic morphological indicators, euploidy rates, and oxidative stress status.

**Conclusion:**

Autologous non-invasively derived USC mitochondria transfer may be an effective strategy to improve embryonic development and metabolism, especially in infertile females with advanced age or repeated pregnancy failure. It provides evidence and possibility for the autologous treatment of infertile females without invasive and ethical concerns.

**Supplementary Information:**

The online version contains supplementary material available at 10.1186/s40659-023-00470-1.

## Introduction

In vitro fertilization/Intracytoplasmic sperm injection (IVF/ICSI) is the mainly used method for infertility therapy, with an international success rate of almost 40%. However, for females with advanced age or diminished ovarian reserve (DOR), the clinical outcome of IVF/ICSI treatment is very poor due to their poor quality of oocytes and embryos, and currently no effective treatment is found. Facing the background of global decline in fertility rates and increasing reproductive age, addressing the issue of infertility in females of advanced age or those experiencing repeated IVF/ICSI failures has become a prominent concern within the field of reproductive medicine[1, 2].

Oocyte aging is closely related to abnormalities in mitochondrial content and function [3]. As the energy metabolism center of cells, mitochondria provide important energy sources for germ cell meiosis, oocyte maturation, fertilization and embryonic development [4]. Among all human cell types, mutural oocytes have the largest number of mitochondria (up to about 200,000) to ensure energy supply [5]. Aged oocytes are frequently accompanied by a series of abnormalities including mitochondrial morphology, mitochondrial DNA (mtDNA) copy number, mtDNA mutation or deletion and biological dysfunction [3, 6, 7], which may further lead to abnormal meiosis in germ cells, increased aneuploidy rates, poor embryo quality and decline in fertility [8].

The emergence of oocyte cytoplasmic mitochondria transfer provides a new approach for solving infertility problems caused by advanced age or diseases. As early as the late twentieth century, a number of European countries tried to improve oocyte quality in the elderly by the simultaneous injection of sperm with 1–5% oocyte cytoplasm from young healthy donors during ICSI, yielding favorable clinical outcomes as reported in NEJM [9–12]. Between 1997 and 2001, about 30 children were born through this technology. However, due to the ethical issues brought about by the third-party genetic materials, the technology was banned by the US Food and Drug Administration (FDA) for clinical use in 2002 and subsequently banned by the UK Human Fertilization and Embryology Authority (HFEA) [13].

Therefore, autologous mitochondrial transfer has recently attracted widespread attention. This technology aims to solve the infertility problem results from advanced age or poor oocyte quality, by extracting mitochondria from autologous cells and co injecting them with single sperm into oocytes during ICSI.

However, studies on this field are relatively scarce, and basic study regarding mitochondrial donor cells is currently lacking. Ongoing clinical investigations are conducted directly on human subjects, without a foundational comprehension of autologous cell mitochondria. Presently, the most used autologous cells for mitochondrial transfer are ovarian stem cells (OSC). Reports regarding its clinical outcomes are inconsistent, including reports of effectiveness in some basic and clinical trials [14–17], while a randomized controlled pilot study yielded no improvement findings [18]. There are doubts about the authenticity of OSC in the adult [19, 20]. Notably, the acquisition of OSC necessitates a surgical procedure involving the removal of substantial portions of ovarian cortex (approximately 3 × 6 mm^3^), potentially inflicting secondary harm upon patients already grappling with compromised ovarian function [18]. Consequently, we believe that currently, the most crucial step is the initial selection of suitable mitochondrial donor cells.

It has been reported in the literature that stem cells including pluripotent stem cells (e.g., ESCs) and adult stem cells (e.g., MSCs). are highly dependent on glycolytic metabolism to maintain stem cell characteristics and self-renewal capacity [21], which is similar to the metabolic pattern of oocytes. This metabolic pattern can reduce mitochondrial production of reactive oxygen species (ROS) and reduce cellular exposure to oxidative stress [22], which are critical for oocyte and stem cell self-renewal, offspring production, and cell fate determination. Besides, due to the strong self-renewal, multi-directional differentiation potential, easy access, and low immunogenicity [23], mesenchymal stromal cells (MSC) are widely used in cell therapeutics, such as adipose and bone marrow-derived mesenchymal stromal cells (ADSC and BMSC). Numerous studies have confirmed that mitochondrial transfer from stem cells to damaged cells plays an important role in stem cell therapy, especially MSC [24]. Therefore, we consider MSCs are potential superior autologous mitochondrial donor cells.

Urine-derived mesenchymal stromal cells (USC) are one type of MSCs derived from kidney epithelium with strong self-renew and multi-directional differentiation potential [25]. Nature protocol reported the first generation of USC from urine samples in 2012 [26]. Since it can be isolated from urine, it possesses the advantages of being easily non-invasively accessed and largely amplified. Many studies consider it as the ideal seed cells for cell therapy and tissue engineering, especially in the urogenital tract (UGT) [27–31].

Our research aims to screen out suitable human autologous oocyte cytoplasmic mitochondrial donor cells by comprehensive assessment of mitochondrial physiology, function and metabolic capacity on a varity of autologous MSCs (including BMSC, ADSC, USC) and ovarian germline GC, as well as to explore the biosafety, effect and mechanism of its mitochondria transfer on human early embryo development.

## Results

### Comparation of mitochondrial morphology, quantity, and function among primary autologous cells

The isolation and culture process of primary USC, GC, BMSC and ADSC was shown in Additional file 3: Fig. S1. After identifying the GC-specific surface marker (Additional file 3: Fig. S2), the in vitro differentiation capacity of USC (Additional file 3: Fig. S3), and the surface-specific markers of MSCs (Additional file 3: Fig. S4), experimental studies were conducted on these four types of primary cells.

**Fig. 1:**
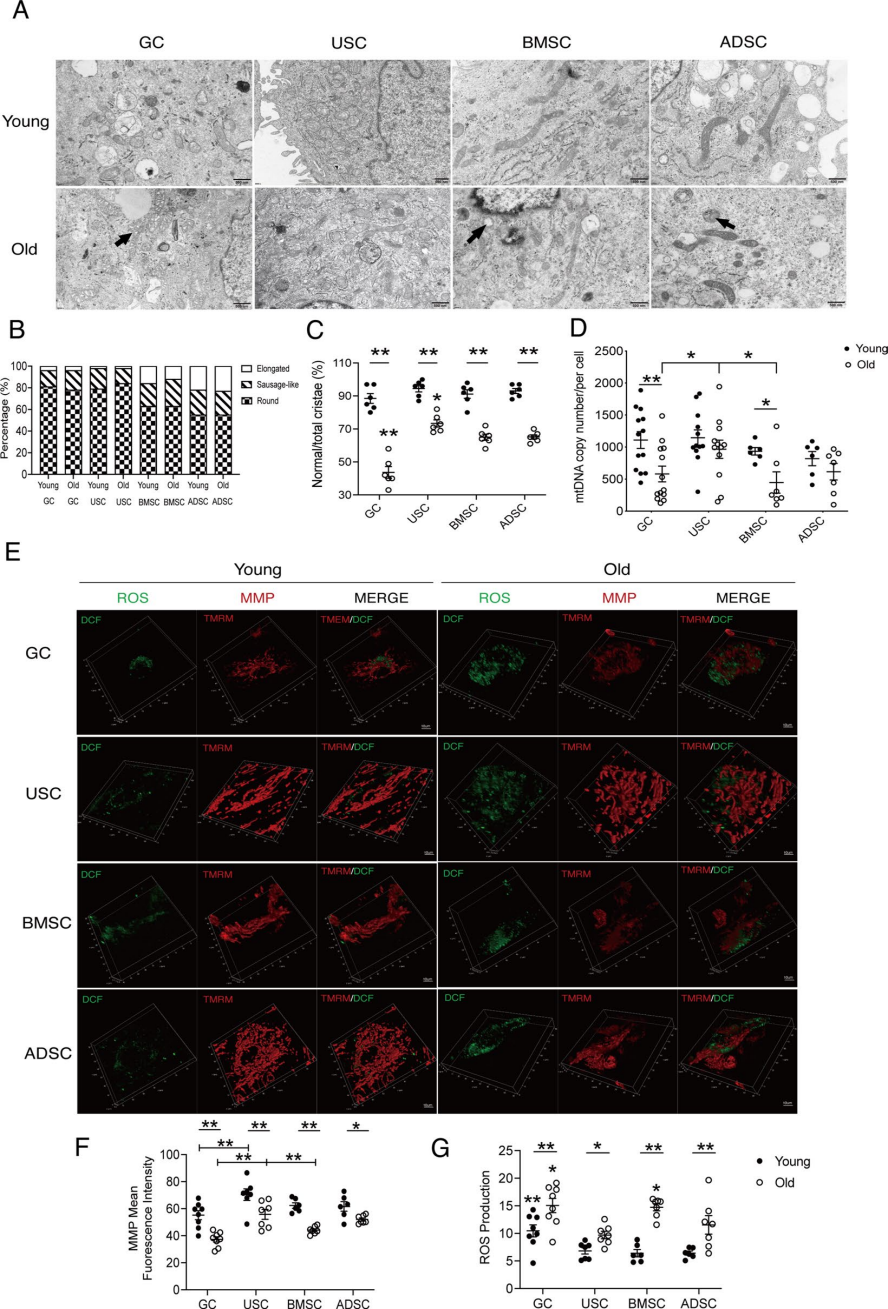
USC mitochondria resemble oocyte morphologically with higher quantities, MMP and lower ROS less affected by age. **A** Mitochondrial morphology of different types of human primary GC and MSCs under transmission electron microscopy (TEM). Arrows indicate mitochondria with abnormal cristae. Scale bars, 500 nm. **B** Percentage of round, sausage-like, elongated shape mitochondria among different cell types. **C** Percentage of mitochondria with normal cristae among different cell types. **D** Absolute quantification of mtDNA copy number by RT-PCR. **E** Mitochondrial membrane potential (MMP, indicated by TMRM) and cytosolic reactive oxygen species (ROS, indicated by DCF) were observed under 3D confocal microscope. Scale bars, 10 μm. **F** The relative abundance of average MMP fluorescence intensity was quantified. **G** The relative abundance of average ROS fluorescence intensity was quantified. Data are shown as means ± SEM. Each scatter represents an independent biological individual. One-way ANOVA, LSD test. *P < 0.05, **P < 0.01

For mitochondrial morphology (Fig. 1A, B), we found that USC and GC demonstrated obviously round-like mitochondria with relative immature cristae, which were similar to oocyte stage. However, BMSC and ADSC mitochondria had more dynamically connected tubular structures, which were sausage-like, elongated in shape with relative mature cristae at the cross sections. All primary cells from the old adults showed strikingly impaired cristae structure and more swollen mitochondria with decreased matrix density (Fig. 1A and C, indicated by arrows), suggesting that advanced age may significantly impair normal cristae structure. Among all types of aged primary cells, GC showed the most severe impaired mitochondrial cristae (vs. USC, *P* < 0.01; Fig. 1C), while USC showed the least severe damage (vs. BMSC and ADSC, *Ps* < 0.05; Fig. 1C).

The results of mtDNA quantification showed that, no significant differences were found among different types of primary cells in the young population. Compared with young adults, mtDNA copy number of the elderly demonstrated a downward trend, among which GC and BMSC reached significance (GC, *P* < 0.01; BMSC, *P* < 0.05; Fig. 1D), but USC still kept at a relative higher level of mtDNA content in the elderly (vs. GC and BMSC, *Ps* < 0.05; Fig. 1D).

MMP was used to indicate mitochondrial activity, as shown in Fig. 1E, we found that MMP of young USC was significantly higher than GC (*P* < 0.01; Fig. 1F). The MMP of the elderly was significantly decreased in all cell types (*Ps* < 0.05; Fig. 1F), but USC still showed a relatively higher MMP than GC and BMSC (*Ps* < 0.01; Fig. 1F).

ROS reflects the level of cytoplasmic oxidative stress. The ROS level of GC was found to be higher than that of MSCs in young adults (*Ps* < 0.01; Fig. 1G). In the elderly, the ROS levels were significantly increased in all cell types (*Ps* < 0.05; Fig. 1G), but GC and BMSC demonstrated relative higher levels than USC and ADSC (*Ps* < 0.05; Fig. 1G).

Together, these results demonstrated that, USC mitochondria resembled oocytes morphologically, and that its mitochondrial content and activity were relatively high and less affected by age than other primary cell types. Besides, its cytosolic oxidative stress is kept at a relatively low level which may be attributed to its immature mitochondrial state, similar to oocytes.

### Comparation of metabolic pattern among primary autologous cells

**Fig. 2:**
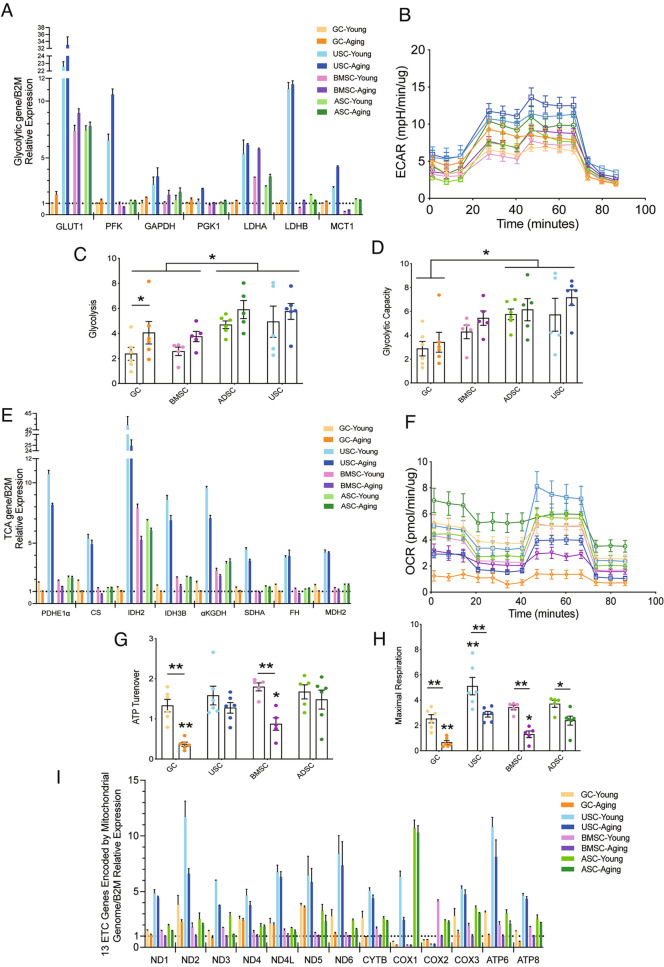
USC shows vigorous metabolism on glycolysis, OXPHOS and mitochondrial genome expression activity. **A** Analysis of glycolytic mRNA expression levels by RT-PCR. **B** Normalized extracellular acidification rate (ECAR) detected by Seahorse XFe96 analyzer. **C** Quantification of glycolysis level. **D** Quantification of glycolytic capacity. **E** Analysis of tricarboxylic acid cycle (TCA) mRNA expression levels by RT-PCR. **F** Normalized oxygen consumption rate (OCR) detected by Seahorse XFe96 analyzer. **G** Quantification of ATP turnover. **H** Quantification of maximal respiration. **I** The mRNA expression level of 13 electron transport chain (ETC) genes encoded by mitochondrial genomes was analyzed by RT-PCR. Data are shown as means ± SEM. Each scatter represents an independent biological individual. One-way ANOVA, LSD test. *P < 0.05, **P < 0.01

We further assessed the metabolic capacity of different types of primary cells. As shown in Fig. 2A, most of the glycolytic genes (*GLUT1, PFK, GAPDH, LDHA*) were highly expressed in MSCs, most notably in USC. Compared with the young group, the expression levels of glycolytic genes of the elderly were significantly up-regulated in all cell types. The extracellular acidification rate (ECAR) detected by Seahorse showed that the glycolytic level and capacity were stronger in USC and ADSC (Fig. 2C–D). Compared with the young group, the elderly showed a trend of increase in glycolysis which was consistent with gene expression results, and GC reached significance in glycolysis level (*P* < 0.05; Fig. 2C).

As shown in Fig. 2E, the highest expression levels of tricarboxylic acid cycle (TCA) genes (*PDHE1α, CS, IDH2, IDH3B, αKGDH, SDHA, FH, MDH2*) were found in USC, while the lowest expression levels were found in GC. Compared with the young, the TCA genes of people with advanced age were significantly down-regulated in all cell types. The results of the oxygen consumption rate (OCR) detected by Seahorse also showed that USC demonstrated the strongest maximal respiratory capacity in the young population (*P* < 0.01; Fig. 2H). With age increasing, the ATP turnover of GC and BMSC decreased (*Ps* < 0.01; Fig. 2G), and the maximal respiration capacity of all cell types decreased significantly (*Ps* < 0.05; Fig. 2H). Among all aged cell types, GC reached the lowest in both ATP turnover and maximal respiration, but USC and ADSC still maintained relatively higher levels (vs. GC, *P* < 0.01; vs. BMSC, *P* < 0.05; Fig. 2 G, H).

The mRNA expression levels of 13 electron transport chain (ETC) genes (*ND1, ND2, ND3, ND4, ND4L, ND5, ND6, CYTB, COX1, COX2, COX3, ATP6, ATP8*) encoded by mitochondrial genomes were analyzed in different types of primary cells (Fig. 2I). Among all cell types, people with advanced age showed significant decreased expression levels compared to the young group, but USC still kept in the highest level regardless of age.

Together, the results showed that the metabolic transformation from oxidative phosphorylation (OXPHOS) to glycolysis is generally occurring in primary cells with increasing age, especially in GC. In addition, the overall cellular metabolism of USC, including glycolysis, OXPHOS, and mitochondrial expression activity, was the most vigorous among all cell types, regardless of age.

### Aged oocytes show aberrant mitochondrial physiology

**Fig. 3:**
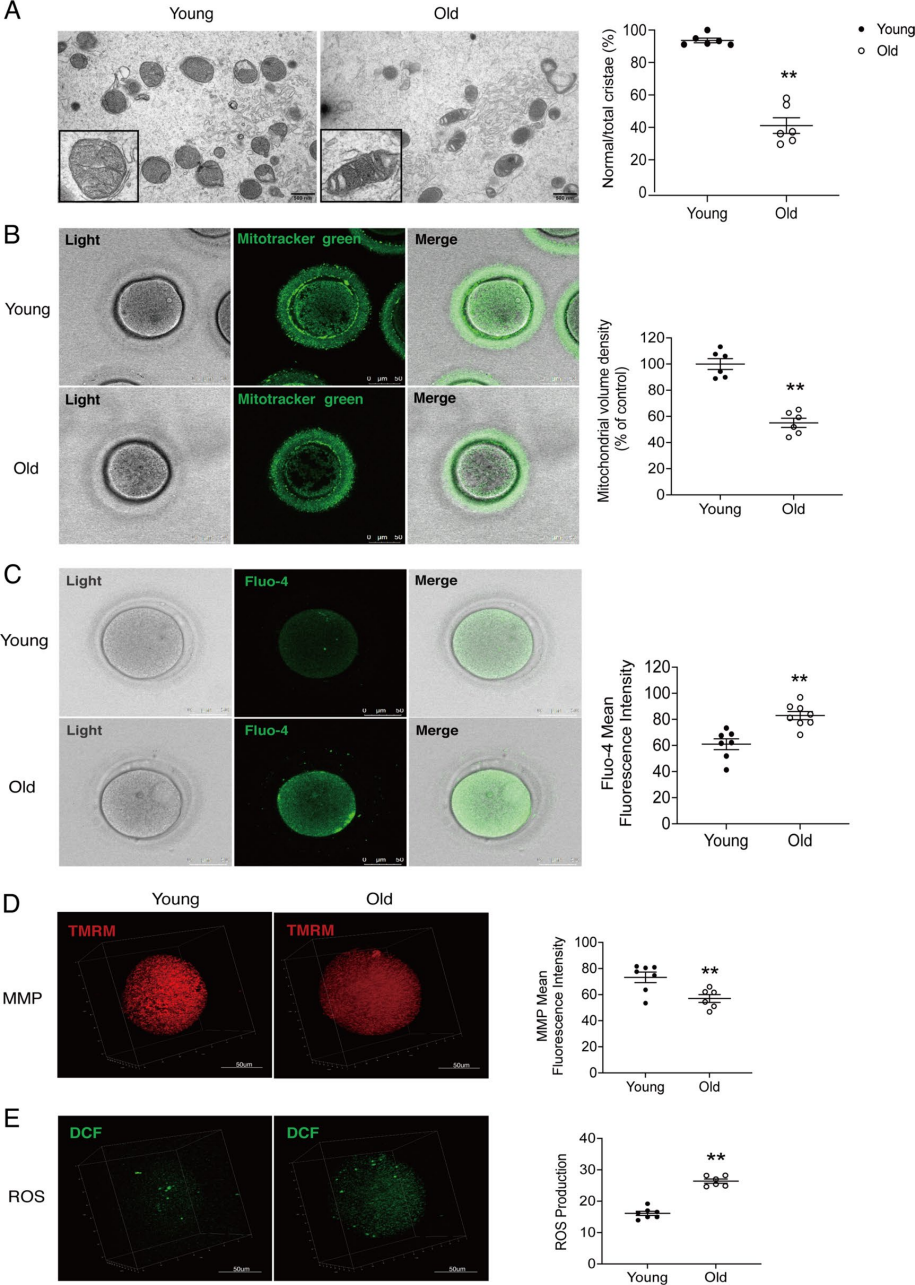
Aberrant phenotype of mitochondrial physiology in aged oocytes. **A** Mitochondrial morphology of human young and aged oocytes under transmission electron microscopy (TEM). Scale bars, 500 nm. **B** Mitochondrial content (indicated by Mitotracker green) was observed under the confocal microscope. Scale bars, 50 μm. **C** Cytosolic Ca^2+^ levels (indicated by Fluo-4) were observed under the confocal microscope. Scale bars, 50 μm. **D** Mitochondrial membrane potential (MMP, indicated by TMRM) were observed under 3D confocal microscope. Scale bars, 50 μm. **E** Cytosolic reactive oxygen species (ROS, indicated by DCF) were observed under 3D confocal microscope. Scale bars, 50 μm. The relative abundance of average fluorescence intensity or area was quantified. Data are shown as means ± SEM. Each scatter represents an independent biological individual. Unpaired two-tailed Student’s t-test. *P < 0.05, **P < 0.01

To examine the effect of age on mitochondria in germ cells, we performed experiments on the oocytes of the young and advanced age. TEM showed that (Fig. 3A), mitochondria of young oocytes were observed to be round in shape, with a high matrix density and immature cristae. However, mitochondria of aged oocytes showed heterogeneous matrix density and cord-like cristae, and the ratio of normal cristae was significantly reduced compared to the young oocytes (*P* < 0.01; Fig. 3A). In addition, Confocal showed that mitochondria content was significantly reduced in aged oocytes (*P* < 0.01; Fig. 3B), accompanied by significantly decreased MMP, increased cytosolic ROS and Ca^2+^ levels (*Ps* < 0.01; Fig. 3C–E). These evidences demonstrate the detrimental effects of age on mitochondrial quantity and quality in oocytes.

In previous studies, we compared various types of autologous primary cells. Among all cell types, USC demonstrated round shaped mitochondria and a relative low ROS level similar to oocytes, as well as abundant mitochondrial content, relative vigorous mitochondrial activity and cellular metabolism.

To further verify the mitochondrial genomic biosecurity of USC, we performed whole mitochondrial genome sequencing on 2 cases of young USC and 2 cases of aged USC (Additional file 3: Fig. S5). We did not observe obvious differences in the number of SNVs and InDels in the D-loop, Gene, tRNA, and rRNA regions of the USC mitochondrial genome, and there was no significant difference in the number of different types of SNVs and InDels in the coding region (Additional file 3: Table S1). Pathogenicity prediction analysis by MutPred and Polyphen-2 HumVar found no pathogenic SNVs and InDels loci with high heterogeneity scores.

Considering USC can also be easily obtained by non-invasive procedure and large-scale expanded, it can act as a superior autologous donor cell for oocyte cytoplasmic mitochondria transfer.

### Autologous non-invasively USC-derived mitochondrial transfer improves mitochondrial content and function of human early embryos, especially in the advanced age

**Fig. 4:**
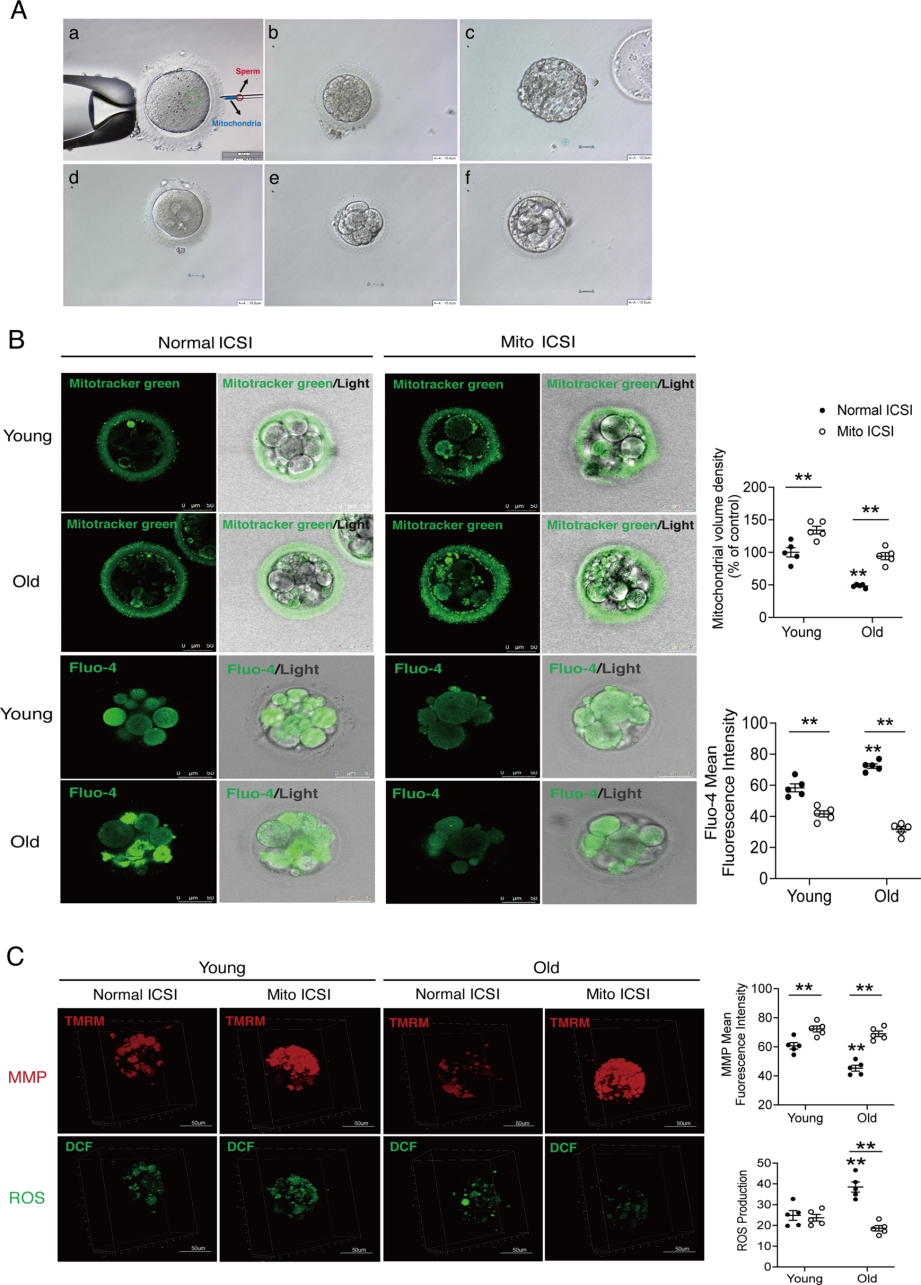
Improvement of mitochondrial content and function in embryos after USC mitochondria transfer. **A** Representative images of early embryo development in the Control and MITO ICSI groups. **a** Co-injection of single sperm with USC-derived mitochondria during ICSI; **b**-**c** Grade IV embryos on day 3 (**b**) and Grade 6CB blastocysts on day 6 (**c**) in the Control group; **d**-**f** Fertilization and 2PN formation on day 1 (**d**), Grade I 8-cells embryos on day 3 (**e**), and Grade 3BC blastocysts on day 5 (**f**) in the MITO ICSI group. **B** Mitochondrial content (indicated by Mitotracker green) and cytosolic Ca^2+^ (indicated by Fluo-4) were observed under confocal microscope. Scale bars, 50 μm. **C** MMP (indicated by TMRM) and cytosolic ROS (indicated by DCF) were observed under 3D confocal microscope. The relative abundance of average fluorescence intensity or area was quantified. Data are shown as means ± SEM. Each scatter represents an independent biological individual. Unpaired two-tailed Student’s t-test. *P < 0.05, **P < 0.01

Mature oocytes (MII stage) were obtained through in-vitro maturation (IVM) culture of immature oocytes (GV or MI stage) from IVF/ICSI patients. Clinical characteristics of donors were listed in Additional file 3: Table S2. Finally, 42 mature oocytes of the young population and 29 mature oocytes of the population with advanced age were included in the following study, and oocytes were further randomized to the corresponding conventional ICSI group and Mito ICSI group. The proportions of GV and MI stage-derived oocytes between the control and treatment groups remained generally consistent. For different age groups, USC mitochondria from young or aged populations were extracted, and transferred during the ICSI process (Fig. 4A, a). For the detailed Mito ICSI operation process, see Additional file 2: Movie S1.

As shown in Fig. 4A, we observed the general embryonic development in the control group (Grade IV embryos on day 3, b; Grade 6CB blastocysts on day 6, c) and the Mito ICSI group (fertilization and 2PN formation on day 1, d; Grade I 8-cells embryos on day 3, e; Grade 3BC blastocysts on day 5, f). Confocal analysis showed that the mitochondrial content of early embryos in the population with advanced age was significantly lower than that in the young, but after USC mitochondrial transfer, the mitochondrial content of both young and aged embryos increased significantly (*Ps* < 0.01; Fig. 4B), revealing the effective mitochondrial transfer process during ICSI. Futher, we analyzed cytoplasmic physiological state and mitochondrial function. Compared with young embryos, aged embryos demonstrated lower MMP, and higher levels of cytosolic ROS and Ca^2+^ (*Ps* < 0.01; Fig. 4B, C). After USC mitochondrial transfer, significant increase of MMP and decrease of Ca^2+^ were observed in both young and aged embryos (*Ps* < 0.01; Fig. 4B, C), however, the improvement was generally greater in the advanced age. Besides, the aged embryos also showed significantly decreased cytosolic ROS after mitochondrial transfer, but no difference was observed in the young embryos (Fig. 4C).

### Autologous non-invasively USC-derived mitochondrial transfer improves morphological development and euploidy rate of human early embryos with advanced age

Morphological evaluation of early embryos is listed in Table 1. Although not statistically significant due to the limited number of available embryos, we observed an upward trend in the rate of 7–10 cell embryos at EDD3 in both of the young and old population after mitochondria transfer, with the young group rose from 9.5% (2/21) to 23.8% (5/21), and the elderly group rose from 26.7% (4/15) to 50.0% (7/14). In addition, in old populations with mitochondria transfer, we also found an upward trend in the rate of good-quality embryos at EDD3 which increased from 20.0% (3/15) to 42.9% (6/14), and the rate of blastocyst formation at EDD5 which increased from 13.3% (2/15) to 21.4% (3/14). Moreover, one high-quality blastocyst (Grade 4BB) was obtained only in the advanced age with mitochondria transfer (1/14).

**Table 1:** Evaluation of embryo morphological and ploidy between the Control and Mito ICSI group

	Young			Old		
	Normal ICSI	Young source Mito ICSI	*P*-value	Normal ICSI	Old source Mito ICSI	*P*-value
Embryo morphological evaluation						
No. of embryo screened	21	21		15	14	
Rate of fertilization (2PN) at EDD1 (%)	71.4 (15/21)	76.2 (16/21)	0.73	73.3 (11/15)	71.4 (10/14)	0.62
Rate of good-quality embryos at EDD3 (%)	4.8 (1/21)	9.5 (2/21)	0.55	20.0 (3/15)	42.9 (6/14)	0.18
Rate of 7–10 cell embryos at EDD3 (%)	9.5 (2/21)	23.8 (5/21)	0.21	26.7 (4/15)	50.0 (7/14)	0.18
Rate of blastocyst formation at EDD5 (%)	9.5 (2/21)	4.8 (1/21)	0.55	13.3 (2/15)	21.4 (3/14)	0.47
Rate of good-quality blastocyst at EDD5 (%)	0.0 (0/21)	0.0 (0/21)	–	0.0 (0/15)	7.1 (1/14)	0.48
Embryo ploidy detection						
No. of embryo screened	10	8		7	9	
Detection rates (%)	50.0 (5/10)	50.0 (4/8)	0.68	42.9 (3/7)	77.8 (7/9)	0.18
Euploid (%)	0.0 (0/5)	0.0 (0/4)	–	0.0 (0/3)	28.6 (2/7)	0.47
Aneuploid (%)						
Segmental aneuploidy (≥ 10 Mbp) (%)	20.0 (1/5)	25.0 (1/4)	0.72	33.3 (1/3)	14.3 (1/7)	0.53
Complex aneuploidy (≥ 3 variations) (%)	60.0 (3/5)	75.0 (3/4)	0.60	66.7 (2/3)	57.1 (4/7)	0.67
Trisomy (%)	0.0 (0/5)	0.0 (0/4)	–	0.0 (0/3)	0.0 (0/7)	–
Monosomy (%)	0.0 (0/5)	0.0 (0/4)	–	0.0 (0/3)	0.0 (0/7)	–
Mosaic (%)	20.0 (1/5)	0.0 (0/4)	0.56	0.0 (0/3)	0.0 (0/7)	–

Data are shown as percentages. Pearson Chi-Square (n ≥ 40) and Fisher’s Exact Test (n < 40)

*PN* pronuclear, *EDD1* the 1st day of embryo development, *EDD3* the 3rd day of embryo development, *EDD5* the 5th day of embryo development, *Mbp* mega base pairs

Interestingly, in the conventional ICSI groups, decreased formation rates of good-quality embryos and 7–10 cell embryos at EDD3, as well as blastocyst formation rates at EDD5, were found in the young population when compared to the advanced age. Actually, clinical immature oocytes obtained from the young and elderly patients have their own developmental problems, which may be related to the aging factor in the population with advanced age, and may also be caused by a number of complex factors in the young population that we do not yet fully understand, despite our inclusion criteria have excluded a range of basic diseases. This phenomenon of improvement in the early embryonic development after mitochondria transfer in the elderly may explain the dominant role of age-mitochondrial related abnormalities in this population, while the young population seems to be associated with more abnormal non-mitochondrial related factors, which needs to be further explored in future studies.

Further, we detected the euploidy rate of early embryos in the Control and the MITO ICSI group of both the young and elderly population (Table 1). Due to the poor quality of the embryos derived from clinically discarded immature oocytes, the average detection rates were around 50%. Encouragingly, consistent with our previous observations in embryo morphology, the highest detection rate was found in the advanced age with mitochondria transfer (77.8%, 7/9). Moreover, among all embryos, 2 euploid embryos were found only in the advanced age with mitochondria transfer (28.6%, 2/7), however, the other groups were all aneuploid or mosaic embryos. Although not statistically significant due to the limited number of available embryos, the results suggest that mitochondria transfer in IVF populations with advanced age may exert favorable effects on the restoration of embryo euploidy. Representative results of euploidy, aneuploidy (segmental aneuploidy, complex aneuploidy), and mosaicism were shown in Additional file 3: Fig. S6.

## Discussion

Our study suggests that, USC can be considered as superior donor cells for autologous oocyte cytoplasmic mitochondria transfer for its advantage of mitochondrial physiology, quantity, activity, metabolism, biosafety, urogenital origin and its non-invasive acquisition. Supplementation of functional mitochondria by USC mitochondria transfer may restore embryonic euploidy and normal embryonic development by improving mitochondrial content, function and metabolism, especially for the population with advanced age or mitochondrial abnormalities.

This is a fact that the associations between cell proliferation, mitochondrial metabolism, and mitochondrial dynamics are quite complex among different stem cell types, and may be highly plastic and context-dependent [32]. Actually, the cellular mitochondrial network is constantly regulated by a continuous cycle of mitochondrial fusion and fission [33]. The dynamic balance between the two is critical for mitochondria to acquire the morphological structure needed to fulfill the specific cellular requirements, as well as to rapidly respond to environmental cues and adapt to bioenergetic needs [34]. Fused, interconnected mitochondrial structures are commonly found in cells that are metabolically active and mainly rely on OXPHOS for energy production. In contrast, non-fused spherical mitochondria are generally more common in quiescent or glycolytic-dominant cells [35]. Many studies have reported that the metabolic pattern of glycolysis as well as the non-fused spherical mitochondria may represent a feature of stemness [32]. One possible reason for this metabolic state of stem cells may be to maintain stem cell homeostasis by reducing mitochondrial metabolism and the production of harmful free radicals, which is similar to the naive mitochondrial morphology and metabolic characteristics of the oocyte stage [22].

In our study, compared with other available autologous cells, USC mitochondria demonstrated non-fused spherical morphology, sufficient quantity, low ROS level, and robust and bivalent metabolism dependent on both glycolysis and OXPHOS. Besides, among all cell types, the mitochondrial number, activity, and metabolic capacity of USC are least affected by age, and related mitochondrial parameters remain at a relatively high level in both the young and the elderly. These findings suggest that the cytoplasmic environment and metabolic requirements of USC mitochondria may be more similar to the oocyte and early embryo stage than other MSCs, which may benefit from its original source of urogenital tract.

After confirming the USC mitochondrial biosafety by whole mitochondrial genome sequencing, early embryos after USC mitochondria transfer show improvements in mitochondrial content, MMP, cytoplasmic Ca^2+^ level, embryo morphological parameters and euploidy rates, with which these changes are more prominent in the elderly population. We also find that embryonic oxidative stress status and ploidy rates are improved in the elderly after USC mitochondria transfer, suggesting that USC mitochondria transfer may be more therapeutically beneficial for the improvement of embryo quality associated with age related mitochondrial abnormalities.

In the field of assisted reproduction, the phenomenon of mitochondrial abnormalities in oocytes from patients with advanced age or DOR has been reported by the literature scatterly [3, 36]. Our previous study showed that mitochondrial morphology, quantity and function of ovarian germline granulosa cells (GC) is severely changed among IVF patients with low prognosis, especially these elderly patients with DOR [37]. Our current results further confirm the fact that, in aged oocytes and early embryos, a series of physiological problems including mitochondrial morphology, mtDNA copy number, MMP, oxidative stress as well as cytosolic Ca^2+^ levels are impaired. Mitochondria-targeted treatment, including both drug therapy and novel techniques, become prospective stratages for IVF patients with advanced age or mitochondrial genetic diseases [38–40]. As early as the end of the twentieth century, heterologous ooplasmic transfer was used to improve oocyte quality in some European countries and achieved a certain degree of therapeutic results [9–11]. More than 30 children were born through this technique, and no abnormal follow-up has been found in the offspring yet [41]. However, its clinical application was prohibited due to possible negative effects mediated by mito-nuclear interactions following allogeneic mitochondrial genome replacement and ethical issues related to third-party genetic materials [13, 42], besides, the basic research on its effect is also quite lacking. Our study provides evidence and possibility for the autologous mitochondria transfer in infertile females without invasive and ethical concerns, and opens up a new and broad prospect in the field of infertility to some extent. However, whether the transfer of autologous mitochondrial genomes might pose risks to the viability and development of early-stage embryos still needs to be evaluated in future studies.

Our study is an in-vitro basic research on human early embryos, and does not involve clinical evaluation after embryo transfer. Therefore, further clinical trials are still needed to confirm its therapeutic effects on clinical pregnancies rates, miscarriage rates and live birth rates.

## Conclusions

Autologous USC-derived mitochondria transfer may serve as a novel modality for the improvement of clinical outcomes in infertile patients with advanced age or unexplained repeated IVF/ICSI failures in the future. It provides evidence and possibility for the autologous treatment of infertile females without invasive and ethical concerns.

## Materials and methods

### Study design

The objective of the present study was to (i) screen out the superior autologous mitochondrial donor cells which was biofunctionally appropriate, easily accessible, highly biosecure and suitable for oocyte cytoplasmic mitochondria transfer, (ii) verify the mitochondrial physiological differences between the young and aged oocytes, and (iii) explore the effect and possible mechanism of superior stem cell-derived mitochondria transfer on early embryonic development among the young and old IVF populations.

Bone marrow, adipose, and urine-derived primary MSC (BMSC, ADSC, USC) and ovarian germline GC were comprehensively assessed for multiple parameters including mitochondrial morphology, mtDNA copy number, mitochondrial activity, metabolic capacity and patterns. Whole mitochondrial genome sequencing was performed to validate the biosecurity of transferred mtDNA. Among the oocytes of the young and old IVF population, we analyzed mitochondrial morphology, mitochondrial content, mitochondrial membrane potential (MMP), cytosolic ROS and Ca^2+^ levels to clarify the differences in potential mitochondrial physiology. Finally, we extracted USC mitochondria from corresponding young and old IVF populations and co-injected them with single sperm during ICSI. The effect of mitochondrial transfer on early embryo development between different age groups was evaluated by embryo morphology, euploidy, mitochondrial content, MMP, cytosolic ROS and Ca^2+^ levels. Please see the detailed experimental methods in the Additional file 1: Methods.

The oocytes used in this study were obtained by in vitro maturation (IVM) culture of discarded immature eggs (GV or MI stage) from IVF/ICSI patients at the Assisted Reproductive Technology (ART) Center of Peking University People’s Hospital (Beijing, China). Mature MII oocytes were divided into young group (< 35) and advanced age group (≥ 35) according to age. Each age group was randomly assigned to the conventional ICSI group and the ICSI with mitochondria group. A total of 71 oocytes available for research were included. Primary GC and USC were isolated and cultured from discarded follicular fluid and urine of IVF/ICSI patients at the ART Center of Peking University People’s Hospital. Primary BMSC and ADSC were donated by the Department of Hematology and Obstetrics of Peking University People’s Hospital. Various types of primary cells were divided into young group (< 35) and advanced age group (≥ 35) according to age, and finally, there are 13 cases of primary young GC, 13 cases of aged GC, 12 cases of young USC, 12 cases of aged USC, 6 cases of young BMSC, 7 cases of aged BMSC, 6 cases of young ADSC, 7 cases of aged ADSC were obtained for research use. All included individuals were excluded from polycystic ovarian syndrome, endometriosis, chromosomal abnormalities or other chronic diseases (e.g., cardiovascular, endocrine, autoimmune diseases, and tumors).

For detailed experimental methods, please refer to Additional file 1: Methods.

### Study approval

This is an in-vitro basic research conducted on human early-stage embryos, with samples sourced from discarded oocytes and oocytes matured through in vitro induction within the ART Center of Peking University People’s Hospital (Beijing, China). The use of these samples was based on patient consent and ethical committee approval by the institutional review board of Peking University People’s Hospital, exclusively for experimental research purposes. No financial interests were involved in the donation process.

### Statistics

Statistical analysis was performed using SPSS 25.0 (IBM). Data were plotted using Prism 9.0 (GraphPad). Unless stated otherwise, data are presented as means ± SEM. Data points represent independent biological samples. Variable differences between the two experimental groups were assessed using paired or unpaired two-tailed Student’s t-test, as indicated in figure legends. One-way analysis of variance (ANOVA) followed by LSD test was used for comparisons of multiple treatment groups. Pearson Chi-Square (n ≥ 40) and Fisher’s Exact Test (n < 40) were used to examine the frequency distribution between the two groups. Shapiro–Wilk and Levene test were used to examine normality, and homogeneity of variance. Data with skewed distributions were transformed logarithmically to approximate normality before analysis. A *P* value of < 0.05 was considered statistically significant.

### Supplementary Information

**Additional file 1:** **Methods.**

**Additional file 2:** **Movie S1. **The operation process of USC-derived mitochondria transfer during ICSI.

**Additional file 3: Fig. S1. **The isolation and culture process of primary USC (**A**), GC (**B**), BMSC (**C**) and ADSC (**D**). Scale bars, 50 μm. **Fig. S2. **Identification of GC-specific surface marker FSHR. **A** Negative control. **B** Primary GC were positive for FSHR expression (stain brown). Scale bars, 50 μm. **Fig. S3. **USC tri-lineage differentiation in vitro. **A** Oil red O staining indicated lipid droplet formation after 21 days of in vitro adipogenic differentiation of USC. Scale bars, 50 μm. **B**, **C** In vitro osteogenic differentiation of USC after 14 days was identified by Alizarin red staining (**B**) and ALP staining (**C**) to indicate calcium nodule formation. Scale bars, 250 μm. **D**–**F** After 28 days of in vitro chondrogenic differentiation of USC, Toluidine blue (**D**), Safranin-O (**E**) and Masson’s trichrome (**F**) stainings revealed the presence of collagen and glycosaminoglycan (GAG) in the extracellular matrix. Scale bars, 50 μm. **Fig. S4. **The positive expressions of MSC surface specific markers in USC (**A**), BMSC (**B**) and ADSC (**C**). Black peaks represented isotype controls and green peaks represented various markers. All primary cells showed CD105 (+), CD73 (+), CD44 (+), HLA-DR (-), CD34 (-), CD45 (-). **Fig. S5. **Whole mitochondrial genome sequencing of 2 pairs of young and old USC.** A** Mitochondrial Circos map of YOUNG USC 1. **B** Mitochondrial Circos map of YOUNG USC 2. **C** Mitochondrial Circos map of OLD USC 1. **D** Mitochondrial Circos map of OLD USC 2. According to the structure of mitochondria, the Circos drawing is carried out according to the statistical depth. The Circos diagram has a total of 4 circles from the outside to the inside. The first ring is the mitochondrial H strand; the second ring is the mitochondrial L strand; in the third ring, the blue part is the coverage depth of each site of the mitochondria; the fourth ring is the position scale of the mitochondria. **Fig. S6.** Representative results of embryo ploidy by SurePlex WGA.** A** Representative result of euploid blastocyst from the Mito-ICSI group. **B** Representative result of segmental aneuploidy (≥ 10 Mbp) with deletion of ch.22q11.1-q13.33. **C** Representative result of complex aneuploidy (≥ 3 variations) with deletion of ch.4p16.3-q35.2, mosaicism at ch.6 and ch.14. **D** Representative result of mosaicism with both the partial duplication and deletion of multiple chromosomes. The black arrows represent the site of abnormal chromosomes. WGA, whole genome amplification** Table S1. **Comparison of USC mitochondrial genome sequencing between the young and old populations. **Table S2. **Clinical characteristics of IVF/ICSI donors of immature oocytes.

## Data Availability

Data and material will be made available by the corresponding author upon request. Whole mtDNA Sequencing data has been archived according to Springer Nature data policy (the BioProject ID PRJNA913803).

## Abbreviations

IVF: In vitro fertilization
ICSI: Intracytoplasmic sperm injection
DOR: Diminished ovarian reserve
mtDNA: Mitochondrial DNA
ROS: Reactive oxygen species
MMP: Mitochondrial membrane potential
FDA: US Food and Drug Administration
HFEA: UK Human Fertilization and Embryology Authority
OSC: Ovarian germline-derived ovarian stem cells
MSC: Mesenchymal stromal cells
ADSC: Adipose-derived mesenchymal stromal cells
BMSC: Bone marrow-derived mesenchymal stromal cells
ESC: Embryonic stem cells
USC: Urine-derived mesenchymal stromal cells
GC: Ovarian germline granulosa cells
UGT: Urogenital tract
IVM: In vitro maturation
ART: Assisted reproductive technology
ECAR: Extracellular acidification rate
TCA: Tricarboxylic acid cycle
OCR: Oxygen consumption rate
ETC: Electron transport chain
OXPHOS: Oxidative phosphorylation
MITO: Mitochondria

## References

[1] Laisk T, Tšuiko O, Jatsenko T, Hõrak P, Otala M, Lahdenperä M (2019). Demographic and evolutionary trends in ovarian function and aging. Hum Reprod Update.

[2] Mascarenhas MN, Flaxman SR, Boerma T, Vanderpoel S, Stevens GA (2012). National, regional, and global trends in infertility prevalence since 1990: a systematic analysis of 277 health surveys. PLoS Med.

[3] May-Panloup P, Boucret L, Chao de la Barca JM, Desquiret-Dumas V, Ferré-L’Hotellier V, Morinière C (2016). Ovarian ageing: the role of mitochondria in oocytes and follicles. Hum Reprod Update.

[4] Van Blerkom J (2011). Mitochondrial function in the human oocyte and embryo and their role in developmental competence. Mitochondrion.

[5] Turnbull DM, Rustin P (2016). Genetic and biochemical intricacy shapes mitochondrial cytopathies. Neurobiol Dis.

[6] Kasapoğlu I, Seli E (2020). Mitochondrial dysfunction and ovarian aging. Endocrinology.

[7] Steffann J, Monnot S, Bonnefont JP (2015). mtDNA mutations variously impact mtDNA maintenance throughout the human embryofetal development. Clin Genet.

[8] Bentov Y, Casper RF (2013). The aging oocyte–can mitochondrial function be improved?. Fertil Steril.

[9] Barritt J, Willadsen S, Brenner C, Cohen J (2001). Cytoplasmic transfer in assisted reproduction. Hum Reprod Update.

[10] Cohen J, Scott R, Schimmel T, Levron J, Willadsen S (1997). Birth of infant after transfer of anucleate donor oocyte cytoplasm into recipient eggs. Lancet.

[11] Cohen J, Alikani M, Garrisi JG, Willadsen S (1998). Micromanipulation of human gametes and embryos: ooplasmic donation at fertilization VIDEO. Hum Reprod Update.

[12] Templeton A (2002). Ooplasmic transfer–proceed with care. N Engl J Med.

[13] Caicedo A, Aponte PM, Cabrera F, Hidalgo C, Khoury M (2017). Artificial mitochondria transfer: current challenges, advances, and future applications. Stem Cells Int.

[14] Woods DC, Tilly JL (2015). Autologous germline mitochondrial energy transfer (AUGMENT) in human assisted reproduction. Semin Reprod Med.

[15] Mobarak H, Heidarpour M, Tsai PJ, Rezabakhsh A, Rahbarghazi R, Nouri M (2019). Autologous mitochondrial microinjection; a strategy to improve the oocyte quality and subsequent reproductive outcome during aging. Cell Biosci.

[16] Oktay K, Baltaci V, Sonmezer M, Turan V, Unsal E, Baltaci A (2015). Oogonial precursor cell-derived autologous mitochondria injection to improve outcomes in women with multiple IVF failures due to low oocyte quality: a clinical translation. Reprod Sci.

[17] Morimoto Y, Gamage USK, Yamochi T, Saeki N, Morimoto N, Yamanaka M (2023). Mitochondrial transfer into human oocytes improved embryo quality and clinical outcomes in recurrent pregnancy failure cases. Int J Mol Sci.

[18] Labarta E, de Los Santos MJ, Herraiz S, Escribá MJ, Marzal A, Buigues A (2019). Autologous mitochondrial transfer as a complementary technique to intracytoplasmic sperm injection to improve embryo quality in patients undergoing in vitro fertilization-a randomized pilot study. Fertil Steril.

[19] Zhang H, Zheng W, Shen Y, Adhikari D, Ueno H, Liu K (2012). Experimental evidence showing that no mitotically active female germline progenitors exist in postnatal mouse ovaries. Proc Natl Acad Sci U S A.

[20] Zhang H, Panula S, Petropoulos S, Edsgärd D, Busayavalasa K, Liu L (2015). Adult human and mouse ovaries lack DDX4-expressing functional oogonial stem cells. Nat Med.

[21] Zhang H, Menzies KJ, Auwerx J (2018). The role of mitochondria in stem cell fate and aging. Development.

[22] Dumollard R, Carroll J, Duchen MR, Campbell K, Swann K (2009). Mitochondrial function and redox state in mammalian embryos. Semin Cell Dev Biol.

[23] Berebichez-Fridman R, Gómez-García R, Granados-Montiel J, Berebichez-Fastlicht E, Olivos-Meza A, Granados J (2017). The holy grail of orthopedic surgery: mesenchymal stem cells-their current uses and potential applications. Stem Cells Int.

[24] Hsu YC, Wu YT, Yu TH, Wei YH (2016). Mitochondria in mesenchymal stem cell biology and cell therapy: from cellular differentiation to mitochondrial transfer. Semin Cell Dev Biol.

[25] Zhang W, Hu J, Huang Y, Wu C, Xie H (2021). Urine-derived stem cells: applications in skin, bone and articular cartilage repair. Burns Trauma.

[26] Zhou T, Benda C, Dunzinger S, Huang Y, Ho JC, Yang J (2012). Generation of human induced pluripotent stem cells from urine samples. Nat Protoc.

[27] Abbas TO, Ali TA, Uddin S (2020). Urine as a main effector in urological tissue engineering-a double-edged sword. Cells.

[28] Sato M, Takizawa H, Nakamura A, Turner BJ, Shabanpoor F, Aoki Y (2019). Application of urine-derived stem cells to cellular modeling in neuromuscular and neurodegenerative diseases. Front Mol Neurosci.

[29] Pavathuparambil Abdul Manaph N, Al-Hawwas M, Bobrovskaya L, Coates PT, Zhou XF (2018). Urine-derived cells for human cell therapy. Stem Cell Res Ther.

[30] Song YT, Li YQ, Tian MX, Hu JG, Zhang XR, Liu PC (2022). Application of antibody-conjugated small intestine submucosa to capture urine-derived stem cells for bladder repair in a rabbit model. Bioact Mater.

[31] Li X, Liao J, Su X, Li W, Bi Z, Wang J (2020). Human urine-derived stem cells protect against renal ischemia/reperfusion injury in a rat model via exosomal miR-146a-5p which targets IRAK1. Theranostics.

[32] Lisowski P, Kannan P, Mlody B, Prigione A (2018). Mitochondria and the dynamic control of stem cell homeostasis. EMBO Rep.

[33] Chen H, Chan DC (2017). Mitochondrial dynamics in regulating the unique phenotypes of cancer and stem cells. Cell Metab.

[34] Campello S, Scorrano L (2010). Mitochondrial shape changes: orchestrating cell pathophysiology. EMBO Rep.

[35] Collins TJ, Berridge MJ, Lipp P, Bootman MD (2002). Mitochondria are morphologically and functionally heterogeneous within cells. Embo J.

[36] Chiang JL, Shukla P, Pagidas K, Ahmed NS, Karri S, Gunn DD (2020). Mitochondria in ovarian aging and reproductive longevity. Ageing Res Rev.

[37] Jiang Z, Shi C, Han H, Wang Y, Liang R, Chen X (2021). Mitochondria-related changes and metabolic dysfunction in low prognosis patients under the POSEIDON classification. Hum Reprod.

[38] Greenfield A, Braude P, Flinter F, Lovell-Badge R, Ogilvie C, Perry ACF (2017). Assisted reproductive technologies to prevent human mitochondrial disease transmission. Nat Biotechnol.

[39] Giannubilo SR, Orlando P, Silvestri S, Cirilli I, Marcheggiani F, Ciavattini A (2018). CoQ10 Supplementation in patients undergoing IVF-ET: the relationship with follicular fluid content and oocyte maturity. Antioxidants.

[40] Zhang Y, Zhang C, Shu J, Guo J, Chang HM, Leung PCK (2020). Adjuvant treatment strategies in ovarian stimulation for poor responders undergoing IVF: a systematic review and network meta-analysis. Hum Reprod Update.

[41] Chen SH, Pascale C, Jackson M, Szvetecz MA, Cohen J (2016). A limited survey-based uncontrolled follow-up study of children born after ooplasmic transplantation in a single centre. Reprod Biomed Online.

[42] Dobler R, Dowling DK, Morrow EH, Reinhardt K (2018). A systematic review and meta-analysis reveals pervasive effects of germline mitochondrial replacement on components of health. Hum Reprod Update.

