# Supplementary material for: Autologous non-invasively derived stem cells mitochondria transfer shows therapeutic advantages in human embryo quality rescue
Source: Biol Res. 2023 Nov 17;56:60. doi: 10.1186/s40659-023-00470-1 (PMC10657142; doi:10.1186/s40659-023-00470-1)
Supplement: Supplementary file 1 — Additional file 1: Methods. [file 40659_2023_470_MOESM1_ESM.docx]

**Supplementary Methods**

**Isolation and culture of human primary GC, USC, BMSC, ADSC**

Human primary GC were collected from the follicle fluid by Percoll density gradient centrifugation as previously described [1]. Briefly, follicle fluid was centrifugated at 400g for 10 min, and sediments were digested with 0.25% trypsin (Gibco) for 10 min. Then, cells were centrifuged at 400g for 5 min, and resuspended in 7 ml PBS (Hyclone). Cell suspensions were slowly added on 7 ml 50% Percoll medium (GE Healthcare) and then centrifuged at 500g for 20 min. The middle GC layer was gently aspirated and washed with PBS. Finally, GC were resuspended in DMEM/F-12 medium (Gibco) supplemented with 1% non-essential amino acid (NEAA) solution (Gibco), 1% GlutaMAX (Gibco), 10% fetal bovine serum (FBS, Gibco), and cultured at 37 °C in a 5% CO_2_ incubator. The medium was changed every other day.

Human USC were isolated and amplificated as described before [2]. Briefly, about 200 ml human fresh urine samples were collected and centrifuged at 1200 rpm for 10 min. Cell sediments were washed with PBS and resuspended in fresh USC medium, and then plated onto 0.1% gelatin-coated 6-well plates and incubated at 37 °C in a 5% CO_2_ incubator. On the third day, nonadherent cells were removed by washing with PBS. Around 5-8 days, clones derived from single cells could be observed under the optical microscope and labeled passage 0 (P0). About 2 weeks, single clones fused and then could be passaged by 0.25% trypsin before over confluence. The USC culture medium was prepared as previous study reported [3], by combining medium A and medium B in a 1:1 ratio. Medium A comprised DMEM/F-12 medium (Gibco) supplemented with 1% NEAA (Gibco), 1% GlutaMAX (Gibco), and 10% FBS (Gibco). Medium B was renal epithelial cell growth medium (REGM BulletKit, CC-3191 & CC-4127, Lonza).

About 3ml fresh bone marrow was collected by heparin anticoagulation tube from healthy hematology individuals, and then added into an equal volume of Ficoll medium (GE Healthcare). After centrifugation at 1800 rpm for 15 min, the layer of mononuclear cells was aspirated and washed by PBS twice. Cell sediments were resuspended in DMEM/F12 medium supplemented with 1% NEAA, 1% GlutaMAX, 10% FBS, and cultured at 37 °C in a 5% CO_2_ incubator. After 48 hours, the medium was completely changed, and the colony formation of adherent cells could be observed under the inverted microscope, which was recorded as P0. The medium was changed every three days and cells were passaged with 0.25% trypsin.

Adipose tissue was obtained from healthy obstetric individuals. Berifly, adipose tissues were washed with PBS repeatedly, and cut into small pieces. Then, an equal volume of 0.75% type I collagenase (Gibco, 17018029) was added to digest in a 37°C shaking incubator for 2 hours. An equal volume of low-glucose DMEM medium (Gibco) supplemented with 1% NEAA, 1% GlutaMAX and 10% FBS was added to stop digestion. After centrifugation at 1200 rpm for 10 min, cell sediments were resuspended in the previously mentioned culture medium, and cultured at 37 °C in a 5% CO_2_ incubator. After 48 hours, the medium was completely changed, and the colony formation of adherent cells could be observed under an inverted microscope, which was recorded as P0. The medium was changed every three days and cells were passaged with 0.25% trypsin.

The primary MSCs were utilized for experimentation at the passage 3, and the passage numbers of both the elderly and youthful cells used remained consistent throughout the experiments.

**Identification of GC and MSCs**

Immunocytochemistry staining was applied to identify the FSHR biomarker of GC. After fixed by 4% paraformaldehyde (PFA) and blocked by 5% BSA, cells were incubated with rabbit anti-human FSHR (1:100; Abcam, ab113421) or PBS as control at 4°C overnight. Next, the HRP-conjugated goat anti-rabbit IgG (1:1000; Abcam, ab6721) was added as a secondary antibody, and the sections were incubated in the dark for 1 hour. Slides were counterstained with hematoxylin. Cells stain brown were considered FSHR positive.

In vitro differentiation potential of USC was detected by osteogenic, adipogenic, and chondrogenic induction. Briefly, when USCs reached 90% confluence, cells were osteogenic lineage induced by osteogenic induction media (Cyagen Biosciences), and adipogenic lineage induced by adipogenic induction media (Gibco, Invitrogen Corp). Alkaline phosphatase staining was performed on day 14. Alizarin Red S staining was utilized to detect calcified matrix deposition at day 21. Oil Red O staining was performed to detect lipid droplets at day 28. Briefly, cells were fixed in 4% PFA for 20 min and then stained with corresponding dyes, and observed under an inverted optical microscope. For chondrogenic induction, about 5 × 10^5^ cells were centrifuged at 150g for 5 min in a sterile 15 ml polypropylene centrifuge tube and incubated overnight to form a high-density cell pellet. The chondrogenic culture medium (Gibco, Invitrogen Corp) was replaced every three days. After 28 days of induction, the pellets were fixed in 4% PFA and the method of sectioning with paraffin was used. The sections at 4 μm thickness were stained with toluidine blue, Safranin O, and Masson trichrome.

Identification of MSC biomarkers of isolated primary USC, BMSC, ADSC was performed by flow cytometry according to the instructions of human MSC biomarker detection kit (Cyagen, HUXMX-09011). Briefly, about 3×10^5^ cells were mixed with 100 μl 0.1%BSA and 2 μl phycoerythrin (PE)-labeled primary antibody CD105, CD73, CD44, HLA-DR, CD34, CD45, and corresponding isotype controls including IgG1, IgG2b. After incubating on ice for 30 min, cells were washed by centrifugation at 250g for 5 min twice. Then, cells were mixed with 100 μl 0.1%BSA and 2 μl of corresponding secondary antibody against the primary antibody. After incubated on ice for 30 min and washing by centrifugation twice. Cells were resuspended in 300 μl 0.1%BSA and immediately detected by Calibur2 flow cytometer (BD Biosciences).

**In vitro maturation (IVM) of immature oocytes**

About 1 hour after oocyte retrieval from IVF/ICSI patients, oocytes were stripped. After evaluation of oocyte maturity, immature oocytes (GV or MI stage) were collected in the cleavage medium (G1-plus, Vitrolife, COOK) and placed in a three-gas incubator with 6% CO_2_, 5% O_2_ for IVM overnight. After 20-21 hours of IVM culture, the first polar body of the oocytes was observed, and the oocytes released the first polar body were included in this study. Oocytes with flat or incomplete polar bodies and multipolar bodies were excluded. Finally, 42 mature oocytes in the young population and 29 mature oocytes in the elderly population were included in this research, and oocytes were further randomized to corresponding conventional ICSI groups and mitochondrial ICSI groups.

**USC mitochondria extraction and the mitochondria transfer during ICSI**

Mitochondria of young and old donor-derived primary USC were extracted by the mitochondria extraction kit (Sigma，MITOISO2). The mitochondrial content in USC was detected by absolute quantitative PCR. Then, based on the cell count, the extracted mitochondrial quantity was calculated. These mitochondria were re-suspended in a specific volume of preservation solution to determine the mitochondrial concentration. Finally, the number of transplanted mitochondria based on the injection volume during transplantation was calculated.

Mitochondrial suspensions were kept on ice before use. ICSI was performed 6-8 h after the first polar body was released. Briefly, single sperm was grabbed and put against the tip of the injection needle (4.5 μm inner side, Sunlight). Then, the injection needle was moved into the mitochondrial suspension droplet, and aspirate mitochondria gently to ensure the volume of mitochondria required for injection (about 1pl). Finally, mitochondria and a single sperm were injected into the matured oocytes. After ICSI, oocytes were cultured in the cleavage medium, and the blastocyst medium (G2-plus, Vitrolife, COOK) was replaced on the third day. Embryo morphologic indices including 2PN fertilization rate, 7-10 cell or good-quality embryo formation rate on the third day, good-quality blastocyst and blastocyst formation rate on the fifth day, were observed and recorded.

**Transmission electron microscopy (TEM)**

Mitochondrial microstructure was observed under TEM. Cells were fixed with 2.5% glutaraldehyde at 4°C overnight. After subsequent dehydration through a graded series of ethanol, samples were embedded in Epon. Ultrathin sections were stained with lead citrate and 2% uranyl acetate, then captured by Philips CM120 TEM.

**Confocal imaging**

Imaging of stained live cells was visualized by confocal microscopy (TCS SP8 STED, Leica). Mitotracker green (Invitrogen), Fluo-4 AM (Beyotime), Tetramethylrhodamine methyl ester (TMRM, Invitrogen), 2',7'-and Dichlorodihydrofluorescein diaceta (DCFH-DA, Invitrogen) were used to indicate mitochondrial content, cytosolic Ca^2+^, MMP and cytosolic ROS levels, respectively. Briefly, cells were pre-planted in confocal dishes and co-incubated with each dye (100 nM Mitotracker green, 2 μM Fluo-4 AM, 100 nM TMRM, 10 μM DCFH-DA, respectively) for 10-30 min. After washing by PBS, medium without serum and phenol red was added into confocal dishes. Mitotracker green fluorescence was detected under the excitation wavelength of 488 nm, and the mitochondrial volume density was calculated by the percentage of mitochondrial area/cytoplasmic area. Fluo-4 fluorescence was detected under the excitation wavelength of 488 nm to determine cytosolic Ca^2+^ levels. TMRM fluorescence was recorded under the excitation wavelength of 525 nm to determine mitochondrial activity. DCFH-DA can cross through the cellular membrane freely and transformed into DCFH in live cells by the cytoplasmic esterase. DCFH could be further oxidized to fluorescent DCF by intracellular ROS. DCF fluorescence was recorded under the excitation wavelength of 488 nm. The mean fluorescence intensity or area was quantified by Fuji Image J software.

**Bioenergetic profiling**

Glycolysis and aerobic respiratory capacity were measured by Seahorse XFe96 Analyzer (Agilent Technologies) as previously described [1, 4]. Primary cells were seeded at 1×10^4^/well in XFe96 microplates (Seahorse Biosciences) and incubated with DMEM/F12 medium for 24 h to achieve 90% confluence on the day of the experiment. To detect mitochondrial aerobic respiratory capacity, the oxygen consumption rate (OCR = pmolesO_2_/min) was analyzed. Briefly, 2 μM oligomycin (oligo), 1 μM FCCP, and a mix of 0.5 μM antimycin A and rotenone (ant/rot) were sequentially added throughout OCR monitoring. Oligomycin and FCCP would stimulate cells to generate the maximum non-phosphorylating respiration and maximum uncoupled respiration, respectively, and the ant/rot mixture would result in the non-mitochondrial oxygen consumption. Related parameters were obtained as follows: ATP turnover = OCR (basal)−OCR (oligo); maximum respiration = OCR (FCCP) − OCR (ant/rot). For the analysis of glycolytic capacity, the extracellular acidification rate (ECAR = mpH/min) was detected. Briefly, 10 mM glucose (glu), an optimal dose of oligomycin, and 50 mM 2-deoxy-D-glucose (2-DG) were sequentially added throughout ECAR monitoring. Oligomycin would contribute to the maximum glycolytic capacity, and 2-DG was to result in the non-glycolytic extracellular acidification. Related parameters were obtained as follows: glycolysis = ECAR (glu) − ECAR (basal); glycolytic capacity = ECAR (oligo) − ECAR (basal). After seahorse analysis, cells were lysised in RIPA buffer (Beyotime) at 4°C for 30 min and centrifuged at 4000 rpm for 40 min. The protein concentration of the supernatant was determined by the BCA protein assay kit (Beyotime), for the further normalization of corresponding parameters.

**Real-time quantitative PCR (RT-PCR)**

MtDNA copy number was quantitied by DNA extraction and RT-PCR procedure as previously described [1, 5]. Briefly, DNA samples of various primary cells were extracted by nucleic acid extraction kit (Beyotime) in accordance with the manufacturer's instructions. RT-PCR was performed by the CFX96 PCR system (Bio-Rad). Briefly, 2 μl DNA was added to a final 10μL reaction mixture including 1 × PCR master mix (Vazyme), double-distilled water, 10 μM of each primer. The quantification cycling protocol was performed as follows: pre-denaturation at 95°C for 5 min, 40 cycles of denaturation at 95°C for 10 s, annealing at 60°C for 30 s, elongation at 95°C for 15 s, and 60°C for 60 s, 95°C for 15 s. Recombinant plasmids containing the β-globin gene and mtDNA ND1 gene were prepared as standard DNA samples.

For analysis of transcript products, RNA samples of various primary cells were extracted by Trizol reagent (Invitrogen). CDNA was obtainted by the reverse transcription kit (TransGen Biotech) according to the manufacturer’s instructions. RT-PCR was performed by the Bio-Rad CFX96 PCR system. Briefly, 1 μl cDNA was added to a final 10 μl reaction mixture including 1 × PCR master mix (Vazyme), double-distilled water, 10 μM of each primer. The quantification cycling protocol was performed as above described. Relative gene expression levels were normalized against endogenous β-2-microglobulin. Data were analyzed after at least two independent experiments.

**Whole mtDNA Sequencing**

Biosecurity of mitochondrial genomes was varified by whole mtDNA sequencing in 2 cases of young USC and 2 cases of aged USC. Briefly, 500 ng of genomic USC DNA was fragmented to a pool (180-220 bp) by Bioruptor Pico (Diagenode), and then each end of the fragments was ligated with adapters (Invitrogen). The Agencourt AMPure XP beads (Beckman) were used to purify adapter-ligated templates, and the purified DNA was amplified by ligation-mediated PCR and then hybridized to a mitochondrial genome panel (iGeneTech) for enrichment. The target region in the panel included the whole mitochondrial genome. The hybridized fragments were bound to Streptavidin Dynabeads (Invitrogen) and then washed with stringent buffers (iGeneTech). The Qubit dsDNA HS Assay Kit (Invitrogen) was used to quantify captured products. Paired-end sequencing was performed by HiSeq Xten instrumentation (Illumina).

**Detection of embryo euploidy**

Detection of embryo euploidy was performed on 34 cases of early embryos (17 cases of the control, 17 cases of the mitochondrial transfer group). Briefly, 3-5 cells from the early embryo were collected in 2 μl PBS as required for optimal lysis of the cells, and immediately frozen in -20℃. Amplification of the whole genome amplification (WGA) was performed using the SurePlex DNA Amplifiction System (Illumina, PR-40-415101-00) as previously described 8[6]. The library was constructed using the TG DNA Library Prep Kit (Veriseq, Illumina, 20029274), and Illumina Nextseq550 was used for sequencing. The sequencing reagent was NextSeq® 500/550 High Output Kit v2 (75 cycles, Illumina, FC-404-2005). High-quality reads were aligned to the NCBI human reference genome (hg19) by BWA software [7]. All steps were performed following manufacturer’s instructions.

**Supplementary references**

1. Jiang Z, Shi C, Han H, Wang Y, Liang R, Chen X, et al. Mitochondria-related changes and metabolic dysfunction in low prognosis patients under the POSEIDON classification. Hum Reprod. 2021;36(11):2904-15. Epub 2021/09/22. doi: 10.1093/humrep/deab203. PubMed PMID: 34545401.

2. Gao X, Jiang Z, Yan X, Liu J, Li F, Liu P, et al. ATF5, a putative therapeutic target for the mitochondrial DNA 3243A > G mutation-related disease. Cell Death Dis. 2021;12(7):701. Epub 2021/07/16. doi: 10.1038/s41419-021-03993-1. PubMed PMID: 34262025; PubMed Central PMCID: PMCPMC8280182.

3. Li X, Liao J, Su X, Li W, Bi Z, Wang J, et al. Human urine-derived stem cells protect against renal ischemia/reperfusion injury in a rat model via exosomal miR-146a-5p which targets IRAK1. Theranostics. 2020;10(21):9561-78. Epub 2020/08/31. doi: 10.7150/thno.42153. PubMed PMID: 32863945; PubMed Central PMCID: PMCPMC7449916.

4. Martino Adami PV, Quijano C, Magnani N, Galeano P, Evelson P, Cassina A, et al. Synaptosomal bioenergetic defects are associated with cognitive impairment in a transgenic rat model of early Alzheimer's disease. J Cereb Blood Flow Metab. 2017;37(1):69-84. Epub 2015/12/15. doi: 10.1177/0271678x15615132. PubMed PMID: 26661224; PubMed Central PMCID: PMCPMC5363729.

5. Chan CC, Liu VW, Lau EY, Yeung WS, Ng EH, Ho PC. Mitochondrial DNA content and 4977 bp deletion in unfertilized oocytes. Mol Hum Reprod. 2005;11(12):843-6. Epub 2006/01/20. doi: 10.1093/molehr/gah243. PubMed PMID: 16421213.

6. Deleye L, De Coninck D, Christodoulou C, Sante T, Dheedene A, Heindryckx B, et al. Whole genome amplification with SurePlex results in better copy number alteration detection using sequencing data compared to the MALBAC method. Sci Rep. 2015;5:11711. Epub 2015/07/01. doi: 10.1038/srep11711. PubMed PMID: 26122179; PubMed Central PMCID: PMCPMC4485032.

7. Li H, Durbin R. Fast and accurate short read alignment with Burrows-Wheeler transform. Bioinformatics. 2009;25(14):1754-60. Epub 2009/05/20. doi: 10.1093/bioinformatics/btp324. PubMed PMID: 19451168; PubMed Central PMCID: PMCPMC2705234.
