# Supplementary figures and images for: Autologous non-invasively derived stem cells mitochondria transfer shows therapeutic advantages in human embryo quality rescue
Source: Biol Res. 2023 Nov 17;56:60. doi: 10.1186/s40659-023-00470-1 (PMC10657142; doi:10.1186/s40659-023-00470-1)

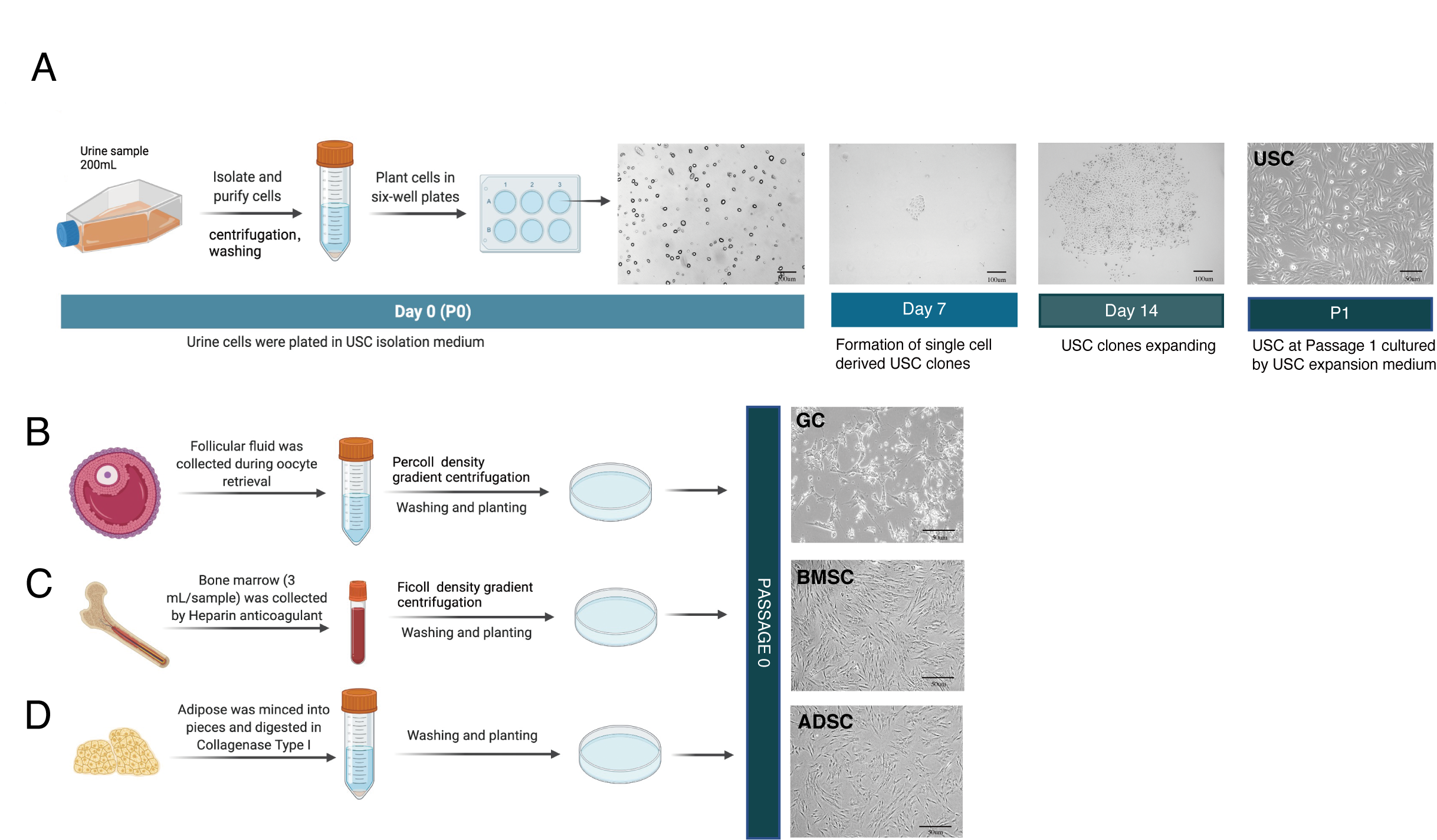

Supplement: Supplementary file 3 — Additional file 3: Fig. S1. The isolation and culture process of primary USC (A), GC (B), BMSC (C) and ADSC (D). Scale bars, 50 μm. Fig. S2. Identification of GC-specific surface marker FSHR. A Negative control. B Primary GC were positive for FSHR expression (stain brown). Scale bars, 50 μm. Fig. S3. USC tri-lineage differentiation in vitro. A Oil red O staining indicated lipid droplet formation after 21 days of in vitro adipogenic differentiation of USC. Scale bars, 50 μm. B, C In vitro osteogenic differentiation of USC after 14 days was identified by Alizarin red staining (B) and ALP staining (C) to indicate calcium nodule formation. Scale bars, 250 μm. D–F After 28 days of in vitro chondrogenic differentiation of USC, Toluidine blue (D), Safranin-O (E) and Masson’s trichrome (F) stainings revealed the presence of collagen and glycosaminoglycan (GAG) in the extracellular matrix. Scale bars, 50 μm. Fig. S4. The positive expressions of MSC surface specific markers in USC (A), BMSC (B) and ADSC (C). Black peaks represented isotype controls and green peaks represented various markers. All primary cells showed CD105 (+), CD73 (+), CD44 (+), HLA-DR (-), CD34 (-), CD45 (-). Fig. S5. Whole mitochondrial genome sequencing of 2 pairs of young and old USC. A Mitochondrial Circos map of YOUNG USC 1. B Mitochondrial Circos map of YOUNG USC 2. C Mitochondrial Circos map of OLD USC 1. D Mitochondrial Circos map of OLD USC 2. According to the structure of mitochondria, the Circos drawing is carried out according to the statistical depth. The Circos diagram has a total of 4 circles from the outside to the inside. The first ring is the mitochondrial H strand; the second ring is the mitochondrial L strand; in the third ring, the blue part is the coverage depth of each site of the mitochondria; the fourth ring is the position scale of the mitochondria. Fig. S6. Representative results of embryo ploidy by SurePlex WGA. A Representative result of euploid blastocyst from the Mito-ICSI gr [file 40659_2023_470_MOESM3_ESM.zip › New folder/Fig. S1.tif]

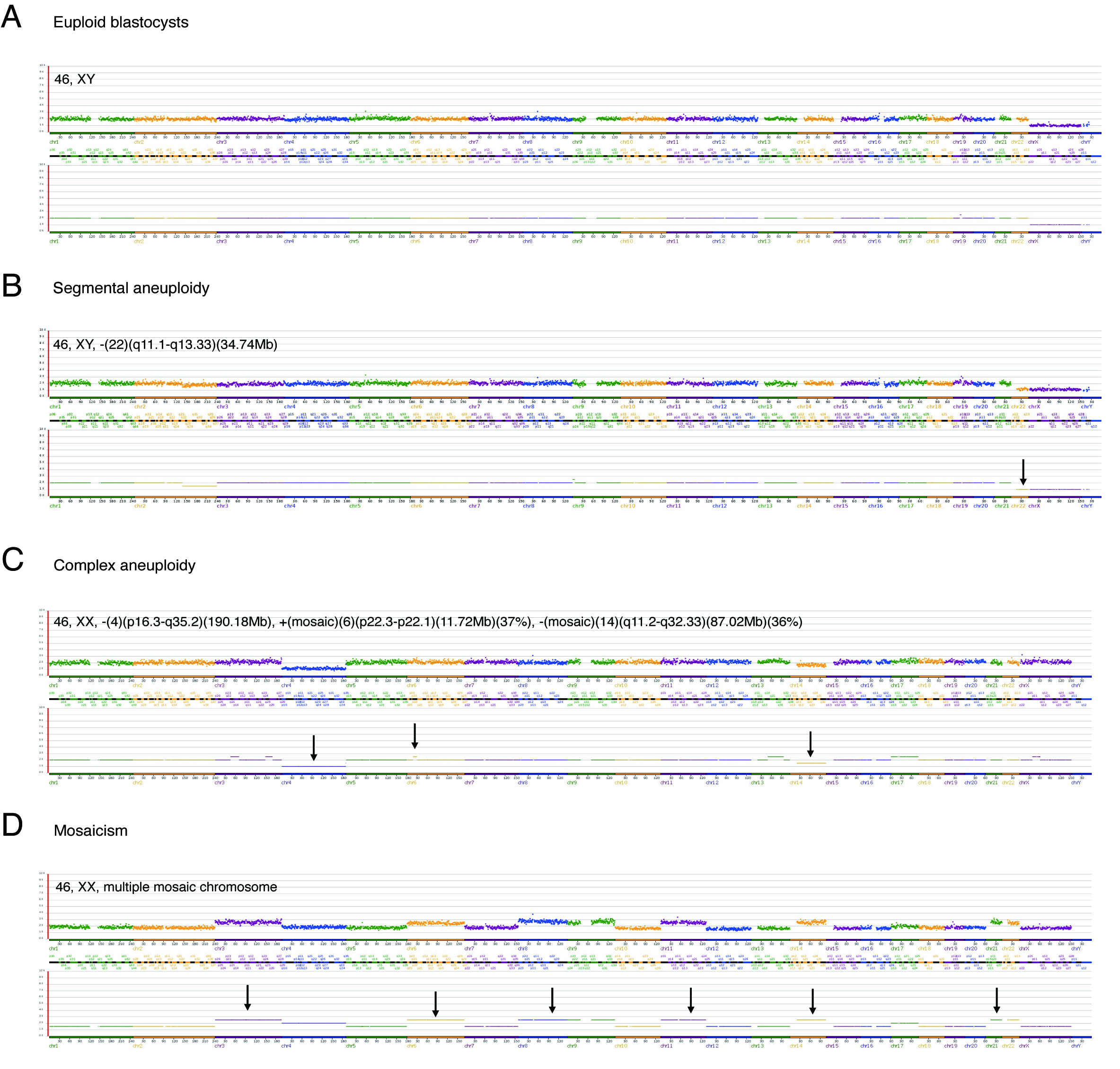

Supplement: Supplementary file 3 — Additional file 3: Fig. S1. The isolation and culture process of primary USC (A), GC (B), BMSC (C) and ADSC (D). Scale bars, 50 μm. Fig. S2. Identification of GC-specific surface marker FSHR. A Negative control. B Primary GC were positive for FSHR expression (stain brown). Scale bars, 50 μm. Fig. S3. USC tri-lineage differentiation in vitro. A Oil red O staining indicated lipid droplet formation after 21 days of in vitro adipogenic differentiation of USC. Scale bars, 50 μm. B, C In vitro osteogenic differentiation of USC after 14 days was identified by Alizarin red staining (B) and ALP staining (C) to indicate calcium nodule formation. Scale bars, 250 μm. D–F After 28 days of in vitro chondrogenic differentiation of USC, Toluidine blue (D), Safranin-O (E) and Masson’s trichrome (F) stainings revealed the presence of collagen and glycosaminoglycan (GAG) in the extracellular matrix. Scale bars, 50 μm. Fig. S4. The positive expressions of MSC surface specific markers in USC (A), BMSC (B) and ADSC (C). Black peaks represented isotype controls and green peaks represented various markers. All primary cells showed CD105 (+), CD73 (+), CD44 (+), HLA-DR (-), CD34 (-), CD45 (-). Fig. S5. Whole mitochondrial genome sequencing of 2 pairs of young and old USC. A Mitochondrial Circos map of YOUNG USC 1. B Mitochondrial Circos map of YOUNG USC 2. C Mitochondrial Circos map of OLD USC 1. D Mitochondrial Circos map of OLD USC 2. According to the structure of mitochondria, the Circos drawing is carried out according to the statistical depth. The Circos diagram has a total of 4 circles from the outside to the inside. The first ring is the mitochondrial H strand; the second ring is the mitochondrial L strand; in the third ring, the blue part is the coverage depth of each site of the mitochondria; the fourth ring is the position scale of the mitochondria. Fig. S6. Representative results of embryo ploidy by SurePlex WGA. A Representative result of euploid blastocyst from the Mito-ICSI gr [file 40659_2023_470_MOESM3_ESM.zip › New folder/Fig. S6.tif]

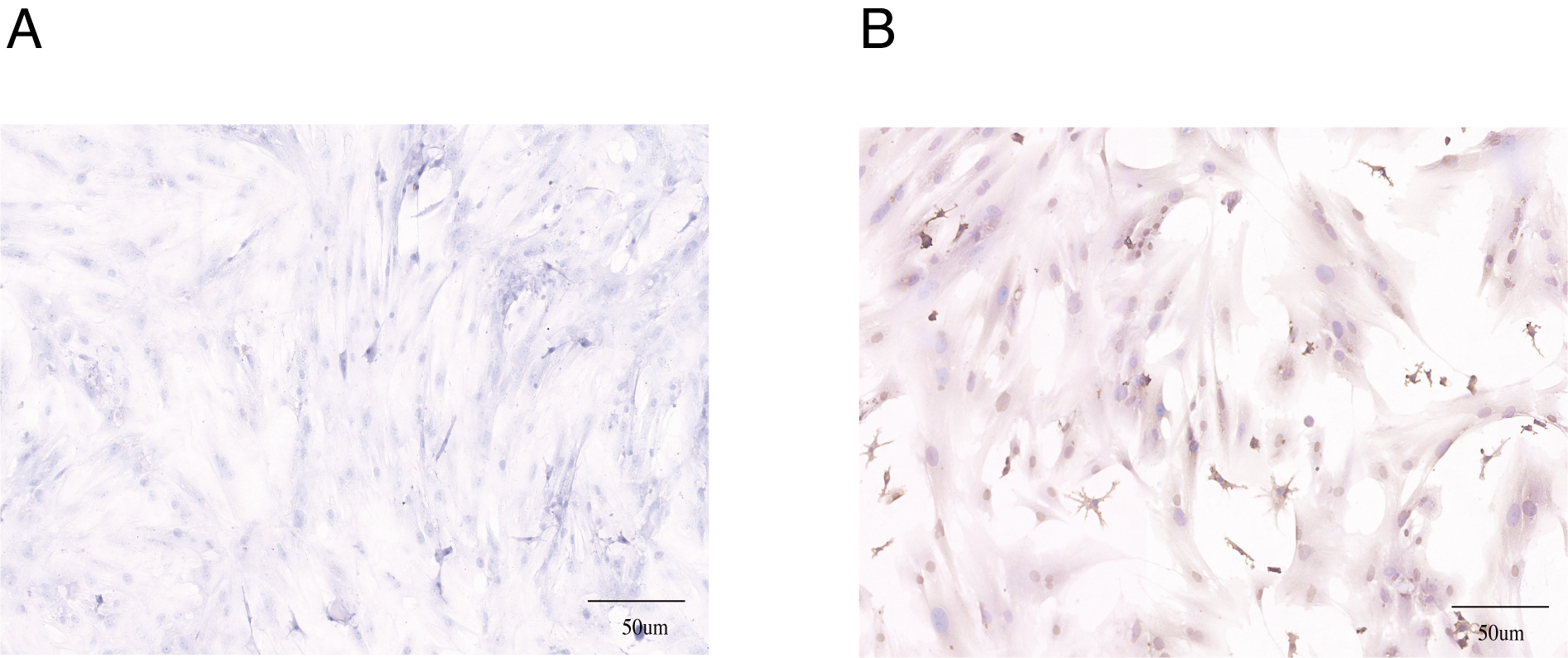

Supplement: Supplementary file 3 — Additional file 3: Fig. S1. The isolation and culture process of primary USC (A), GC (B), BMSC (C) and ADSC (D). Scale bars, 50 μm. Fig. S2. Identification of GC-specific surface marker FSHR. A Negative control. B Primary GC were positive for FSHR expression (stain brown). Scale bars, 50 μm. Fig. S3. USC tri-lineage differentiation in vitro. A Oil red O staining indicated lipid droplet formation after 21 days of in vitro adipogenic differentiation of USC. Scale bars, 50 μm. B, C In vitro osteogenic differentiation of USC after 14 days was identified by Alizarin red staining (B) and ALP staining (C) to indicate calcium nodule formation. Scale bars, 250 μm. D–F After 28 days of in vitro chondrogenic differentiation of USC, Toluidine blue (D), Safranin-O (E) and Masson’s trichrome (F) stainings revealed the presence of collagen and glycosaminoglycan (GAG) in the extracellular matrix. Scale bars, 50 μm. Fig. S4. The positive expressions of MSC surface specific markers in USC (A), BMSC (B) and ADSC (C). Black peaks represented isotype controls and green peaks represented various markers. All primary cells showed CD105 (+), CD73 (+), CD44 (+), HLA-DR (-), CD34 (-), CD45 (-). Fig. S5. Whole mitochondrial genome sequencing of 2 pairs of young and old USC. A Mitochondrial Circos map of YOUNG USC 1. B Mitochondrial Circos map of YOUNG USC 2. C Mitochondrial Circos map of OLD USC 1. D Mitochondrial Circos map of OLD USC 2. According to the structure of mitochondria, the Circos drawing is carried out according to the statistical depth. The Circos diagram has a total of 4 circles from the outside to the inside. The first ring is the mitochondrial H strand; the second ring is the mitochondrial L strand; in the third ring, the blue part is the coverage depth of each site of the mitochondria; the fourth ring is the position scale of the mitochondria. Fig. S6. Representative results of embryo ploidy by SurePlex WGA. A Representative result of euploid blastocyst from the Mito-ICSI gr [file 40659_2023_470_MOESM3_ESM.zip › New folder/Fig.S2.tif]

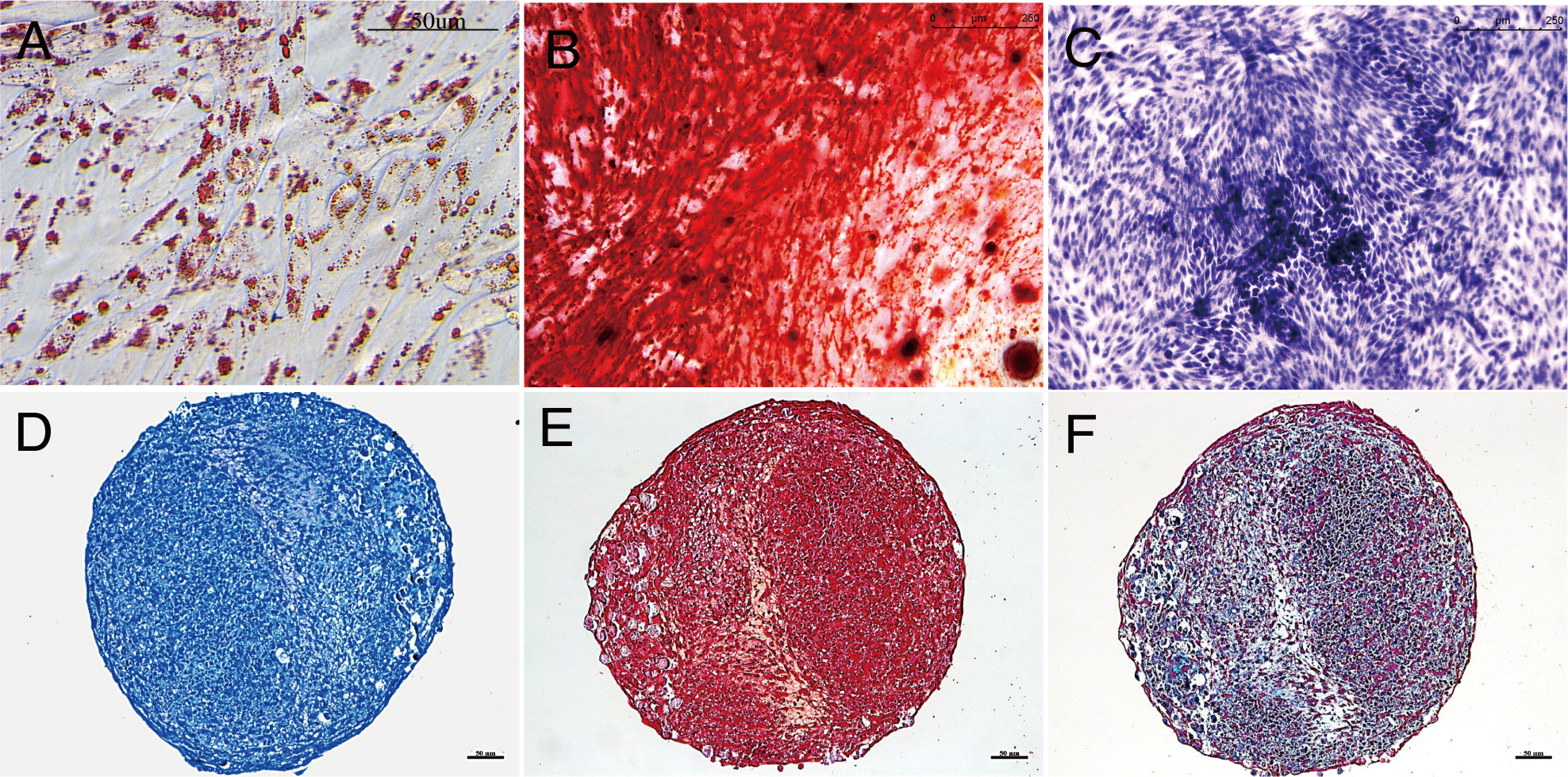

Supplement: Supplementary file 3 — Additional file 3: Fig. S1. The isolation and culture process of primary USC (A), GC (B), BMSC (C) and ADSC (D). Scale bars, 50 μm. Fig. S2. Identification of GC-specific surface marker FSHR. A Negative control. B Primary GC were positive for FSHR expression (stain brown). Scale bars, 50 μm. Fig. S3. USC tri-lineage differentiation in vitro. A Oil red O staining indicated lipid droplet formation after 21 days of in vitro adipogenic differentiation of USC. Scale bars, 50 μm. B, C In vitro osteogenic differentiation of USC after 14 days was identified by Alizarin red staining (B) and ALP staining (C) to indicate calcium nodule formation. Scale bars, 250 μm. D–F After 28 days of in vitro chondrogenic differentiation of USC, Toluidine blue (D), Safranin-O (E) and Masson’s trichrome (F) stainings revealed the presence of collagen and glycosaminoglycan (GAG) in the extracellular matrix. Scale bars, 50 μm. Fig. S4. The positive expressions of MSC surface specific markers in USC (A), BMSC (B) and ADSC (C). Black peaks represented isotype controls and green peaks represented various markers. All primary cells showed CD105 (+), CD73 (+), CD44 (+), HLA-DR (-), CD34 (-), CD45 (-). Fig. S5. Whole mitochondrial genome sequencing of 2 pairs of young and old USC. A Mitochondrial Circos map of YOUNG USC 1. B Mitochondrial Circos map of YOUNG USC 2. C Mitochondrial Circos map of OLD USC 1. D Mitochondrial Circos map of OLD USC 2. According to the structure of mitochondria, the Circos drawing is carried out according to the statistical depth. The Circos diagram has a total of 4 circles from the outside to the inside. The first ring is the mitochondrial H strand; the second ring is the mitochondrial L strand; in the third ring, the blue part is the coverage depth of each site of the mitochondria; the fourth ring is the position scale of the mitochondria. Fig. S6. Representative results of embryo ploidy by SurePlex WGA. A Representative result of euploid blastocyst from the Mito-ICSI gr [file 40659_2023_470_MOESM3_ESM.zip › New folder/Fig.S3.tif]

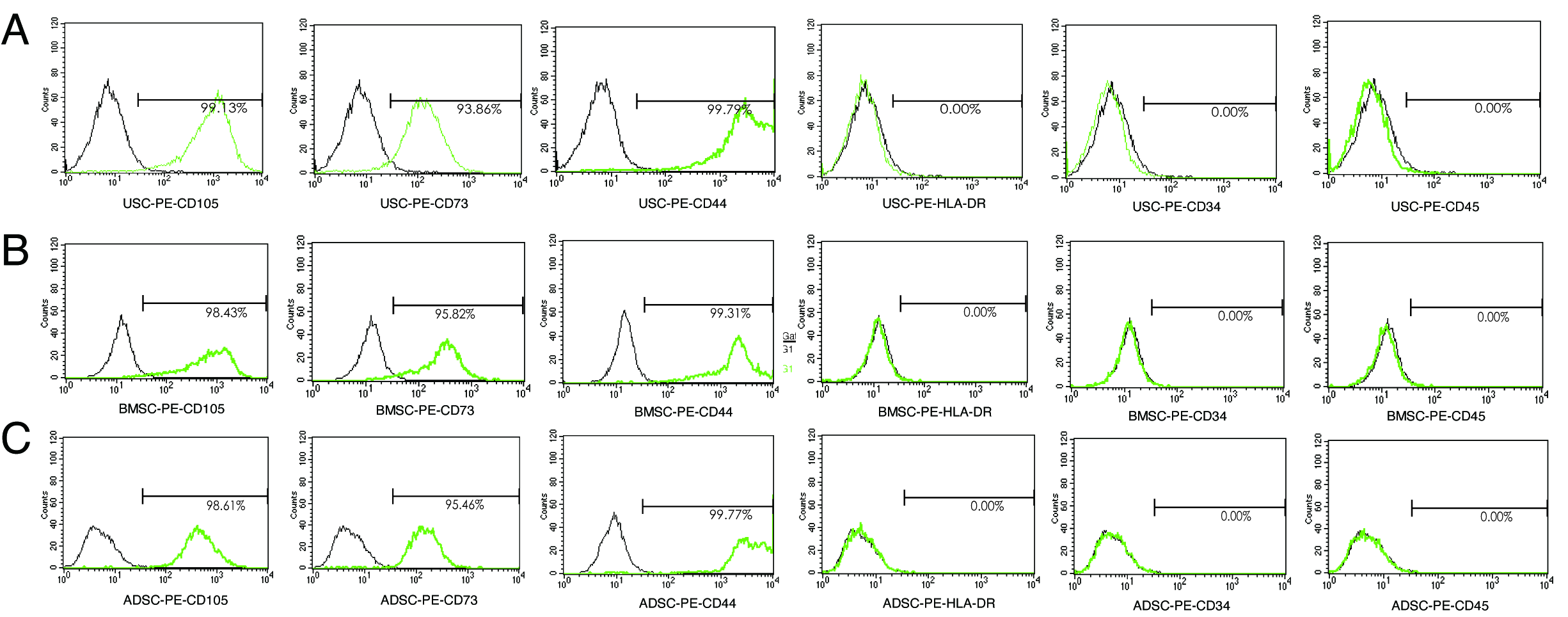

Supplement: Supplementary file 3 — Additional file 3: Fig. S1. The isolation and culture process of primary USC (A), GC (B), BMSC (C) and ADSC (D). Scale bars, 50 μm. Fig. S2. Identification of GC-specific surface marker FSHR. A Negative control. B Primary GC were positive for FSHR expression (stain brown). Scale bars, 50 μm. Fig. S3. USC tri-lineage differentiation in vitro. A Oil red O staining indicated lipid droplet formation after 21 days of in vitro adipogenic differentiation of USC. Scale bars, 50 μm. B, C In vitro osteogenic differentiation of USC after 14 days was identified by Alizarin red staining (B) and ALP staining (C) to indicate calcium nodule formation. Scale bars, 250 μm. D–F After 28 days of in vitro chondrogenic differentiation of USC, Toluidine blue (D), Safranin-O (E) and Masson’s trichrome (F) stainings revealed the presence of collagen and glycosaminoglycan (GAG) in the extracellular matrix. Scale bars, 50 μm. Fig. S4. The positive expressions of MSC surface specific markers in USC (A), BMSC (B) and ADSC (C). Black peaks represented isotype controls and green peaks represented various markers. All primary cells showed CD105 (+), CD73 (+), CD44 (+), HLA-DR (-), CD34 (-), CD45 (-). Fig. S5. Whole mitochondrial genome sequencing of 2 pairs of young and old USC. A Mitochondrial Circos map of YOUNG USC 1. B Mitochondrial Circos map of YOUNG USC 2. C Mitochondrial Circos map of OLD USC 1. D Mitochondrial Circos map of OLD USC 2. According to the structure of mitochondria, the Circos drawing is carried out according to the statistical depth. The Circos diagram has a total of 4 circles from the outside to the inside. The first ring is the mitochondrial H strand; the second ring is the mitochondrial L strand; in the third ring, the blue part is the coverage depth of each site of the mitochondria; the fourth ring is the position scale of the mitochondria. Fig. S6. Representative results of embryo ploidy by SurePlex WGA. A Representative result of euploid blastocyst from the Mito-ICSI gr [file 40659_2023_470_MOESM3_ESM.zip › New folder/Fig.S4.tif]

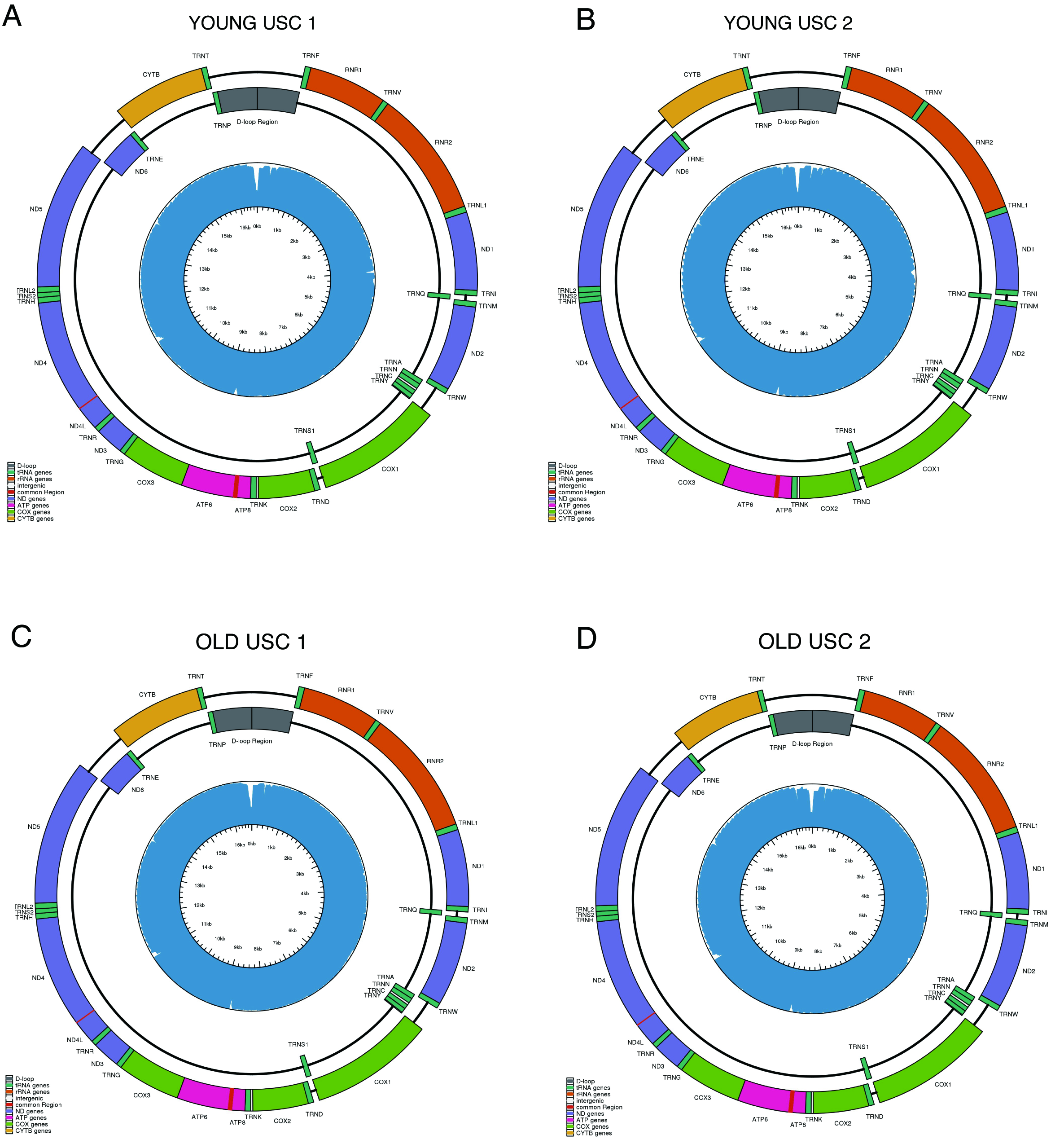

Supplement: Supplementary file 3 — Additional file 3: Fig. S1. The isolation and culture process of primary USC (A), GC (B), BMSC (C) and ADSC (D). Scale bars, 50 μm. Fig. S2. Identification of GC-specific surface marker FSHR. A Negative control. B Primary GC were positive for FSHR expression (stain brown). Scale bars, 50 μm. Fig. S3. USC tri-lineage differentiation in vitro. A Oil red O staining indicated lipid droplet formation after 21 days of in vitro adipogenic differentiation of USC. Scale bars, 50 μm. B, C In vitro osteogenic differentiation of USC after 14 days was identified by Alizarin red staining (B) and ALP staining (C) to indicate calcium nodule formation. Scale bars, 250 μm. D–F After 28 days of in vitro chondrogenic differentiation of USC, Toluidine blue (D), Safranin-O (E) and Masson’s trichrome (F) stainings revealed the presence of collagen and glycosaminoglycan (GAG) in the extracellular matrix. Scale bars, 50 μm. Fig. S4. The positive expressions of MSC surface specific markers in USC (A), BMSC (B) and ADSC (C). Black peaks represented isotype controls and green peaks represented various markers. All primary cells showed CD105 (+), CD73 (+), CD44 (+), HLA-DR (-), CD34 (-), CD45 (-). Fig. S5. Whole mitochondrial genome sequencing of 2 pairs of young and old USC. A Mitochondrial Circos map of YOUNG USC 1. B Mitochondrial Circos map of YOUNG USC 2. C Mitochondrial Circos map of OLD USC 1. D Mitochondrial Circos map of OLD USC 2. According to the structure of mitochondria, the Circos drawing is carried out according to the statistical depth. The Circos diagram has a total of 4 circles from the outside to the inside. The first ring is the mitochondrial H strand; the second ring is the mitochondrial L strand; in the third ring, the blue part is the coverage depth of each site of the mitochondria; the fourth ring is the position scale of the mitochondria. Fig. S6. Representative results of embryo ploidy by SurePlex WGA. A Representative result of euploid blastocyst from the Mito-ICSI gr [file 40659_2023_470_MOESM3_ESM.zip › New folder/Fig.S5.tif]
